# Nucleation‐Controlled Doping of II–VI Semiconductor Nanocrystals Mediated by Magic‐Sized Clusters

**DOI:** 10.1002/smsc.202400300

**Published:** 2024-11-15

**Authors:** Seunghyun Ji, Hafiz Ghulam Abbas, Seo Young Kim, Hyo Cheol Lee, Kyunghoon Lee, Shi Li, Seungho Choe, Hyungju Ahn, Stefan Ringe, Jiwoong Yang

**Affiliations:** ^1^ Department of Energy Science and Engineering Daegu Gyeongbuk Institute of Science and Technology (DGIST) Daegu 42988 Republic of Korea; ^2^ Department of Chemistry Korea University Seoul 02841 Republic of Korea; ^3^ Energy Science and Engineering Research Center Daegu Gyeongbuk Institute of Science and Technology (DGIST) Daegu 42988 Republic of Korea; ^4^ Pohang Accelerator Laboratory Pohang University of Science and Technology (POSTECH) Pohang 37673 Republic of Korea

**Keywords:** 2D nanocrystals, doping, magic‐sized clusters, nucleation‐controlled doping, semiconductor nanocrystals

## Abstract

Doping quantum‐confined semiconductor nanocrystals offers an effective way to tailor their unique properties. However, the inherent challenges of nanoscale doping processes, such as the low probability of successful doping, have hindered their practical applications. Nucleation‐controlled doping has emerged as a potential solution, but a comprehensive mechanistic understanding of this process is lacking. Herein, the nucleation‐controlled doping process facilitated by magic‐sized cluster intermediates is elucidated. This approach enables the synthesis of 2D ZnSe quantum nanoribbons with two distinct doping sites. Remarkably, the identity of the dopants plays a critical role in determining the chemical pathways of nucleation‐controlled doping. Substitutional doping of magic‐sized clusters with Mn^2+^ ions leads to successful substitutional doping of the final 2D nanocrystals. Conversely, Co^2+^ ions, initially occupying substitutional positions in the magic‐sized cluster intermediates, relocate to alternative sites, such as interstitial sites, in the final nanocrystals. First‐principle calculations of dopant formation energies support these experimental findings, demonstrating the thermodynamic favorability of specific dopant site preferences. Moreover, a consistent tendency is observed in CdSe nanocrystals, suggesting that the proposed doping mechanism is generally applicable to II–VI semiconductors. This study will advance the controlled synthesis of various doped semiconductor nanocrystals using nucleation‐controlled doping processes.

## Graphical abstract

Nucleation‐controlled doping through the magic‐sized cluster intermediates is studied, which results in the successful synthesis of 2D ZnSe quantum nanoribbons with different doping sites. The findings, applicable to II–VI semiconductors, significantly enhance the understanding on the underlying mechanism of nanoscale doping processes.

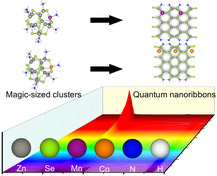

## 1 Introduction

Doping, the intended incorporation of functional impurities into semiconductor materials, is a crucial method for controlling the physical properties of host semiconductors. Specifically, doping of quantum‐confined semiconductor nanocrystals^[^ ^1^, ^2^, ^3^, ^4^, ^5^, ^6^, ^7^, ^8^, ^9^, ^10^, ^11^, ^12^, ^13^, ^14^, ^15^ ^]^ provides an additional avenue to finely tune their characteristics, complementing their unique size‐^[^ ^16^, ^17^, ^18^, ^19^, ^20^, ^21^, ^22^, ^23^ ^]^ and shape‐dependent^[^ ^24^, ^25^, ^26^, ^27^, ^28^, ^29^ ^]^ properties. Conventionally, nanoscale doping has been known to involve the adsorption of impurity atoms onto the nanocrystal surface during the growth stage.^[^ ^30^, ^31^, ^32^, ^33^, ^34^, ^35^ ^]^ Consequently, doping quantum‐confined semiconductor nanocrystals presents greater challenges compared to their bulk counterparts, as the probability of the surface adsorption becomes lower with decreasing nanocrystal size. Nucleation‐controlled doping has emerged as a promising approach for effectively introducing dopants into strongly quantum‐confined CdSe nanocrystals.^[^ ^7^, ^36^, ^37^ ^]^ This method entails the incorporation of dopants into seeds or intermediates, such as magic‐sized clusters,^[^ ^38^, ^39^, ^40^, ^41^, ^42^, ^43^, ^44^, ^45^, ^46^, ^47^, ^48^, ^49^, ^50^ ^]^ at the prenucleation stage, followed by the subsequent formation of nanocrystals while maintaining doping. Notably, this strategy has successfully yielded nanocrystals with high doping concentrations, reaching up to ≈10%.^[^ ^7^, ^36^ ^]^ Nonetheless, fundamental insights into this process (e.g., chemical pathways, failure mechanism, and so on) are missing and its applicability to different materials such as heavy‐metal‐free semiconductors remains largely unexplored.

On the other hand, shape‐controlled synthesis of quantum‐confined semiconductor nanocrystals has been regarded as a fascinating issue.^[^ ^2^, ^10^, ^20^ ^]^ Among various materials, 2D nanocrystals are to be in the limelight because of their exceptional properties, including strong photoluminescence (PL) with an extremely narrow emission linewidth (≈10 nm) resulting from their atomically uniform thickness.^[^ ^51^, ^52^, ^53^, ^54^, ^55^, ^56^, ^57^, ^58^, ^59^, ^60^ ^]^ However, it is difficult to synthesize 2D semiconductor nanocrystals compared to 0D or 1D counterparts because of their intrinsic crystal structure.^[^ ^55^, ^56^ ^]^ For instance, materials with isotropic crystals structure (e.g., cubic) tend to form 0D nanocrystals, while materials with anisotropic crystal structures (e.g., hexagonal) preferentially lead to the synthesis of 1D nanocrystals, facilitated by the high surface energy of the facet perpendicular to the *c*‐axis. Combined with these inherent difficulties, controlling the doping process during the synthesis of 2D semiconductor nanocrystals is extremely challenging. Thus, previous studies have predominantly been limited to Mn^2+^ doping of Cd‐based 2D nanocrystals.^[^ ^7^, ^36^, ^37^ ^]^

Herein, we investigate the magic‐sized cluster‐mediated nucleation‐controlled doping process, leading to the successful synthesis of doped 2D ZnSe quantum nanoribbons. Our findings indicate that the dopant species significantly influence the doping pathways. Specifically, the formation of Mn‐doped (ZnSe)_13_ magic‐sized clusters results in substitutional Mn^2+^ doping in ZnSe quantum nanoribbons, whereas Co^2+^ dopants in (ZnSe)_13_ magic‐sized clusters escape substitutional sites and migrate to alternative positions in the final nanocrystals. Density functional theory (DFT) calculations support these experimental observations, suggesting that the stable doping location is thermodynamically dependent on the chemical nature of the dopants. This shows that even when intermediates are substitutionally doped, the final products may adopt different doping modes. Importantly, similar behaviors are observed in CdSe nanocrystals, demonstrating the broad applicability of our findings to II–VI semiconductor nanocrystals. This study advances our understanding of the nanoscale doping process and paves the way for the development of advanced synthesis techniques for a variety of doped semiconductor nanocrystals.

## 2 Results and Discussion

**Figure 1:**
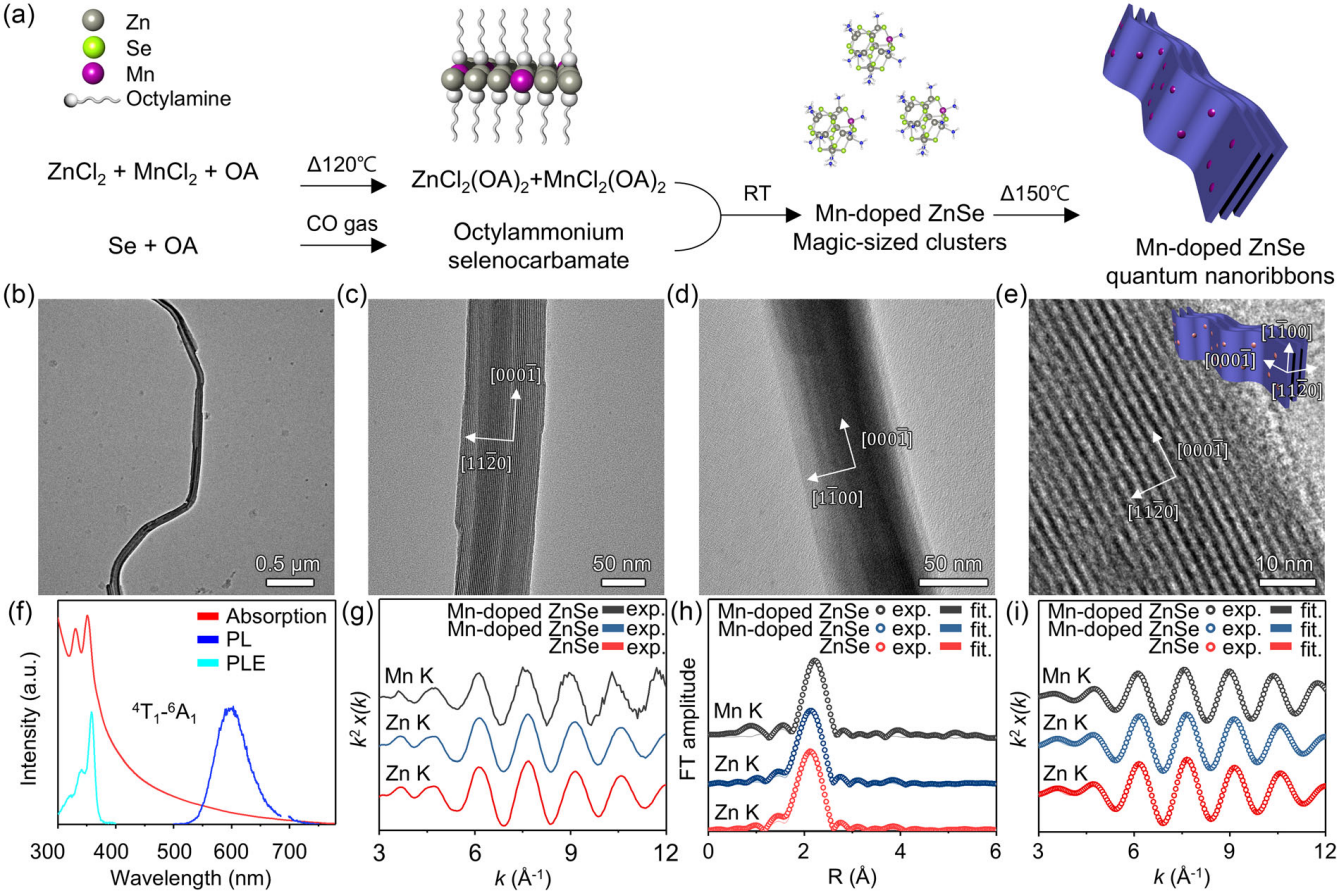
Synthesis and characterization of 2D Mn‐doped ZnSe quantum nanoribbons. a) Schematic showing the synthesis of Mn‐doped ZnSe quantum nanoribbons. b) Low‐resolution, c,d) side‐view, and e) high‐resolution TEM images of Mn‐doped ZnSe quantum nanoribbons (*x*_Mn_ = 12%). f) Absorption, PL, and PLE spectra of Mn‐doped ZnSe quantum nanoribbons (*x*_Mn_ = 7%). g) Experimental *k* ^2^‐weighted Zn and Mn *K*‐edge EXAFS oscillations, h) Fourier‐transformed EXAFS spectra with their fittings based on tetrahedral symmetry, and i) Fourier‐filtered EXAFS spectra (*x*_Mn_ = 12%).

In this study, we selected the doping and synthesis of ZnSe nanocrystals as our model system. It is important to note that previous demonstrations of nucleation‐controlled doping via magic‐sized cluster intermediates have been primarily limited to the synthesis of Mn‐doped CdSe nanocrystals.^[^ ^7^, ^36^ ^]^ To synthesize doped ZnSe quantum nanoribbons, we employed a Lewis acid–base reaction between metal halide–alkylamine complexes and *n*‐octylammonium selenocarbamate (**Figure** **1**a and Experimental Section). It is worth noting that, unlike CdSe quantum nanoribbons, which are typically synthesized at a lower temperature of ≈70 °C,^[^ ^7^ ^]^ the growth of ZnSe quantum nanoribbons requires a growth temperature of ≈150 °C. Deviations from this temperature range result in the unsuccessful synthesis of ZnSe nanoribbons (Figure S1, Supporting Information). The detailed formation pathways of these quantum nanoribbons are discussed later in this article.

Transmission electron microscopy (TEM) images show the 2D morphology of Mn‐doped ZnSe quantum nanoribbons (Figure 1b–d), which closely resemble those of CdSe quantum nanoribbons.^[^ ^7^ ^]^ These quantum nanoribbons exhibit highly elongated lengths along the [$000\bar{0}$] direction, extending to several tens of micrometers (Figure 1b), with a relatively uniform width of ≈65 nm (Figure 1c). They have an extremely uniform thickness of 1.5 nm and form lamellar assemblies due to the strong van der Waals interactions between quantum nanoribbons (Figure 1d,e). Fast Fourier transform analysis (Figure S2, Supporting Information) confirms that Mn‐doped ZnSe quantum nanoribbons possess a hexagonal wurtzite structure with lattice contraction along the lateral direction of the 2D nanocrystals, which is attributed to high surface tension along their atomically flat basal planes.^[^ ^7^, ^52^ ^]^ Importantly, we can produce undoped ZnSe quantum nanoribbons using a similar experimental method by simply excluding the Mn precursor. The shape (including the thickness) and crystal structure of undoped ZnSe quantum nanoribbons closely resemble those of Mn‐doped ZnSe quantum nanoribbons (Figure S3, Supporting Information).

Optical spectroscopy further verifies the successful synthesis of Mn‐doped ZnSe quantum nanoribbons. The absorption spectrum of Mn‐doped ZnSe quantum nanoribbons is almost similar to that of undoped ZnSe quantum nanoribbons (Figure S4, Supporting Information), displaying two sharp peaks at 331 and 351 nm (Figure 1f). These peaks indicate strong transitions resulting from the strong quantum confinement effect arising from the atomically uniform and ultrathin thickness of Mn‐doped ZnSe quantum nanoribbons. The observed splitting of heavy and light hole‐excitonic transitions is one of the representative characteristic features of 2D II–VI semiconductor nanocrystals. Besides, the absorption signal observed beyond 400 nm is attributed to light scattering caused by the lamellar structure of the quantum nanoribbons, which are passivated by short octylamine ligands.^[^ ^36^, ^61^ ^]^ After ligand exchange with oleylamine, which partially disrupts the lamellar assemblies, the scattering tail is significantly reduced, confirming that this scattering effect originates from the lamellar structure (Figure S5, Supporting Information).

The PL spectrum shows the internal manganese transition (^4^T_1_–^6^A_1_) at ≈600 nm, providing evidence for the successful substitutional doping of Mn^2+^ ions in the tetrahedral sites of the host materials.^[^ ^36^, ^62^ ^]^ The position of peaks in the PL excitation (PLE) spectrum aligns well with that of the absorption spectrum, further supporting that the emission is originating from the Mn^2+^ ions in the lattice sites of Mn‐doped ZnSe quantum nanoribbons. X‐ray absorption fine structure (EXAFS) analyses confirm that Mn^2+^ ions occupy tetrahedral sites (Figure 1g–i and Table S1, Supporting Information). The Mn^2+^ doping is additionally verified through energy‐dispersive X‐ray spectroscopy (EDS) elemental analysis (Figure S6 and S7, Supporting Information). The final doping concentration of Mn^2+^ ions in the products can be adjusted by controlling the initial ratio between MnCl_2_ and ZnCl_2_ (Figure S8 and S9, Supporting Information).

**Figure 2:**
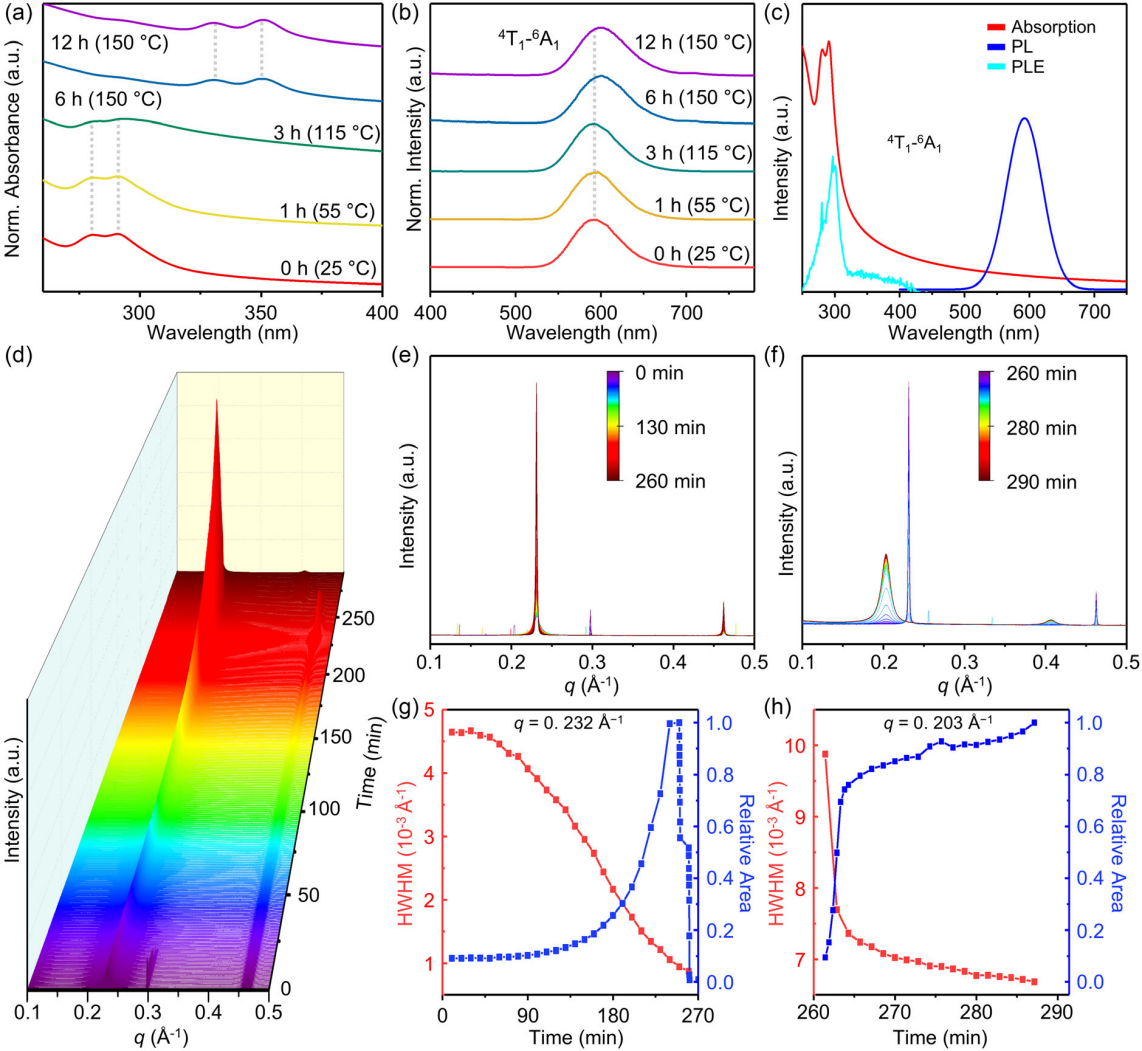
Observation on the formation of 2D Mn‐doped ZnSe quantum nanoribbons. a) Normalized time‐dependent absorption and b) PL spectra of a series of aliquots during the synthesis of Mn‐doped ZnSe quantum nanoribbons (*x*_Mn_ = 7%). c) Absorption, PL, and PLE spectra of Mn‐doped (ZnSe)_13_ magic‐sized clusters (*x*_Mn_ = 6%). d) In situ SAXS patterns depicting the formation of 2D Mn‐doped ZnSe quantum nanoribbons (*x*_Mn_ = 12%). e) Detailed SAXS patterns of the first‐order reflections of lamellar assemblies of Mn‐doped (ZnSe)_13_ magic‐sized clusters (*x*_Mn_ = 13%) and f) Mn‐doped ZnSe quantum nanoribbons (*x*_Mn_ = 12%). Relative SAXS peak area and linewidth (HWHM) of g) the magic‐sized cluster assemblies (*q* = 0.232 Å^−1^) and h) the quantum nanoribbon assemblies (*q* = 0.203 Å^−1^) as a function of reaction time.

To investigate the doping process of Mn‐doped ZnSe quantum nanoribbons, we analyzed the optical spectra of a series of aliquots taken from the reaction mixture during the quantum nanoribbon synthesis (**Figure** **2**a,b). Detailed absorption spectra and TEM images of the aliquots are provided in Figure S10–S12, Supporting Information. In the early stage of the reaction (0 and 2 h in Figure 2a), two peaks at 281 and 291 nm were observed in the absorption spectra, which can be assigned to characteristic transitions of (ZnSe)_13_ magic‐sized clusters (Figure S13, Supporting Information).^[^ ^56^ ^]^ This assignment is further supported by laser desorption/ionization mass spectrometry (Figure S14, Supporting Information). As the reaction proceeded, these peaks associated with the magic‐sized clusters disappeared, and two strong and sharp peaks emerged, corresponding to the heavy and light hole‐excitonic transitions of ZnSe quantum nanoribbons.

Throughout the entire reaction process, PL spectra consistently exhibited the internal manganese transition (^4^T_1_–^6^A_1_, Figure 2b). This offers direct evidence that Mn^2+^ ions were in the tetrahedral sites of the host materials,^[^ ^63^, ^64^ ^]^ confirming that their substitutional doping was maintained. The redshift in the PL peak of the internal manganese transition (from 590 to 600 nm) is attributed to an increase in the Mn—Se bond length during the transformation from the Mn‐doped (ZnSe)_13_ clusters to quantum nanoribbons (Table S1–S4, Supporting Information), a process that will be discussed in detail later in this article. Measurements of absorption, PL, and PLE spectra on the separately synthesized Mn‐doped (ZnSe)_13_ magic‐sized clusters (for experimental details, see Experimental Section) further confirm the successful substitutional doping of (ZnSe)_13_ magic‐sized clusters (Figure 2c). Collectively, these results suggest that the successful substitutional doping of the final nanocrystals is attributable to the substitutional doping of magic‐sized clusters, in line with the nucleation doping process observed in Mn‐doped CdSe quantum nanoribbons.^[^ ^7^, ^36^ ^]^

To gain deeper insights into how the substitutional Mn^2+^ doping of magic‐sized clusters leads to the doping of ZnSe quantum nanoribbons, we carried out in situ small‐angle X‐ray scattering (SAXS) analysis (for experimental details, see Figure S15 and Supporting Methods, Supporting Information). Figure 2d presents the overall in situ SAXS patterns over a period of ≈300 min, during which the magic‐sized clusters evolve into quantum nanoribbons. The initial reaction time profiles (0–260 min) display a series of peaks, indicating the formation of lamellar assemblies of Mn‐doped (ZnSe)_13_ magic‐sized clusters (Figure 2e and S16, Supporting Information). The peak at ≈0.30 Å^−1^ is attributed to *n*‐octylammonium selenocarbamate.^[^ ^61^ ^]^ The position (*q*) of the first‐order reflection (≈0.23 Å^−1^) is corresponding to the interlayer spacing (*d*) of ≈2.71 nm, which is consistent with ex situ SAXS and TEM analyses of Mn‐doped (ZnSe)_13_ magic‐sized clusters (Figure S17 and S18, Supporting Information).

As the reaction progressed, an additional series of reflections emerged (Figure 2f), with the first‐order reflection at 0.203 Å^−1^, which is attributed to the lamellar assemblies of ZnSe quantum nanoribbons (Figure S19, Supporting Information). Representative data from the period when the ZnSe quantum nanoribbon assemblies began to form are presented in Figure S20, Supporting Information, indicating that these changes likely occurred between 2 and 4 h of the reaction time, as shown in Figure 2a. Concurrently, the intensity of the first‐order reflection peak associated with the nanocluster‐lamellar assemblies decreased (Figure 2g), while that of the Mn‐doped ZnSe quantum nanoribbon assemblies increased (Figure 2h). Importantly, a rapid increase in peak area related to the nanoribbon assemblies was observed when both nanocluster‐lamellar and nanoribbon‐lamellar assemblies coexisted; however, this increase slowed considerably once the peaks for the lamellar assemblies of magic‐sized clusters disappeared.

These observations suggest that the transformation from Mn‐doped (ZnSe)_13_ magic‐sized clusters to Mn‐doped ZnSe nanoribbons is the primary pathway for the nanoribbon formation, despite the experimental challenges in directly tracing the clusters during the later stages of the synthesis. It is important to note that the disappearance of the SAXS peaks for the lamellar assemblies of the magic‐sized clusters does not necessarily imply the complete absence of these clusters; rather, it indicates that their lamellar assemblies are no longer forming. This could be due to most clusters being consumed during nanoribbon nucleation, resulting in an insufficient number of the magic‐sized clusters to form lamellar assemblies.^[^ ^61^ ^]^ Additionally, the narrowing of the half‐width at half‐maximum (HWHM) of the first‐order peaks for the Mn‐doped ZnSe quantum nanoribbon assemblies indicates the formation of increasingly ordered lamellar assemblies over time, which also contributes to the increased reflection peak intensity of the nanoribbon assemblies.

These findings are further corroborated by TEM analysis of intermediate species during the synthesis (Figure S12, Supporting Information), which shows consistent trends. At the reaction time of 4 h, significant elongation of the Mn‐doped ZnSe nanoribbons was already evident, and further elongation was challenging to observe with extended reaction time. Instead, the lamellar assemblies of these nanoribbons appeared to become more ordered after 4 h, which is consistent with the changes in HWHM observed in the in situ SAXS measurements. Overall, these findings suggest that Mn‐doped (ZnSe)_13_ magic‐sized clusters transform into Mn‐doped ZnSe quantum nanoribbons within the lamellar assemblies while maintaining substitutional Mn^2+^ doping.

**Figure 3:**
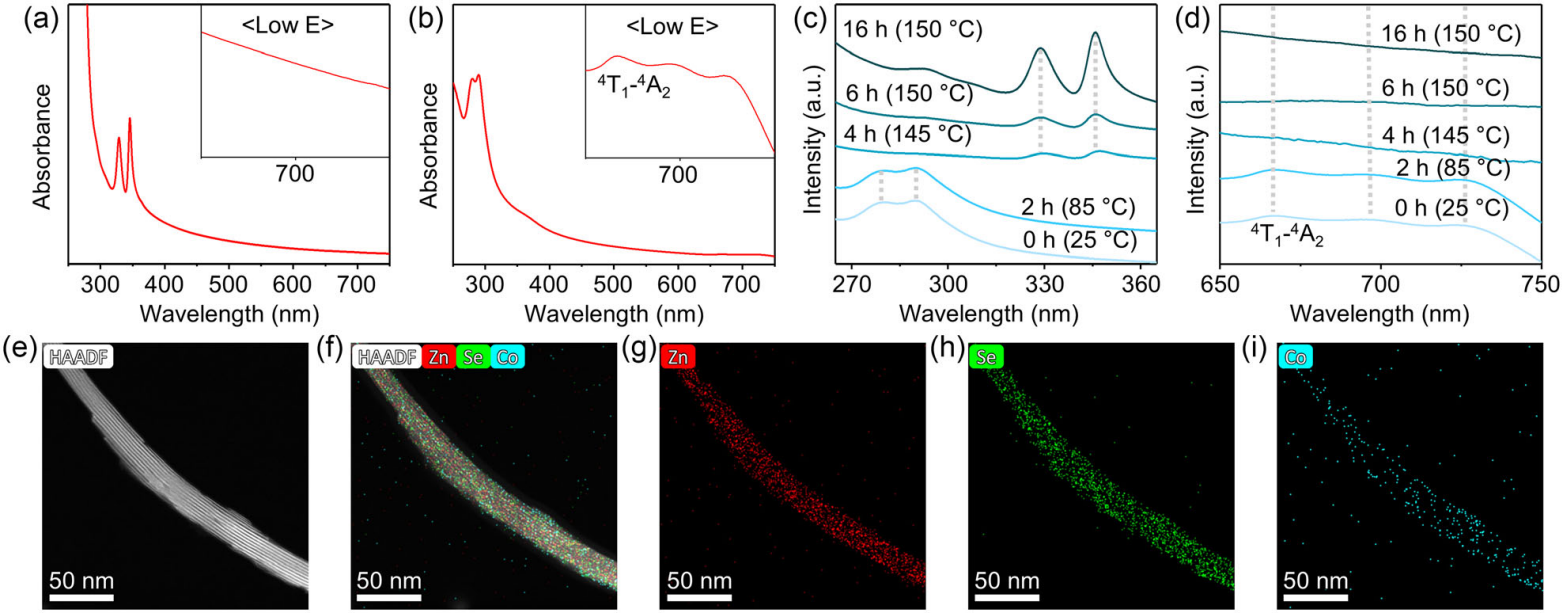
Synthesis of Co‐doped ZnSe quantum nanoribbons. Absorption spectra of a) Co‐doped ZnSe quantum nanoribbons (*x*_Co_ = 5%) and b) Co‐doped (ZnSe)_13_ magic‐sized clusters (*x*_Co_ = 7%). c) Time‐dependent absorption spectra around band‐edge transition of Co‐doped ZnSe quantum nanoribbons (*x*_Co_ = 5%) and d) ^4^T_1_–^4^A_2_ ligand field transition of Co^2+^ ions, measured from a series of aliquots during the synthesis. The first recorded data at room temperature are denoted as 0 min. e–i) High‐angle annular dark‐field scanning transmission electron microscopy (HAADF‐STEM) and EDS elemental mapping images of Co‐doped ZnSe quantum nanoribbons.

To understand how the choice of dopant species affects the chemical pathways of doping, we attempted to synthesize ZnSe quantum nanoribbons using Co(halide)_2_–alkylamine complexes instead of Mn(halide)_2_–alkylamine complexes (**Figure** **3**, see Experimental Section for experimental details). TEM images confirm that the synthesized quantum nanoribbons possess an overall shape similar to that of undoped or Mn‐doped quantum nanoribbons, with an ultrathin and uniform thickness of 1.5 nm (Figure S21, Supporting Information). The absorption spectrum of Co‐doped ZnSe quantum nanoribbons is also nearly identical to that of ZnSe quantum nanoribbons (Figure 3a). All these results corroborate that the host materials, ZnSe quantum nanoribbons, were successfully synthesized with the addition of Co precursors.

As we analyzed the Mn‐doped (ZnSe)_13_ magic‐sized clusters, we separately synthesized Co‐doped (ZnSe)_13_ magic‐sized clusters for detailed analysis (Figure 3b). The band‐edge transitions of Co‐doped (ZnSe)_13_ magic‐sized clusters resemble those of undoped or Mn‐doped (ZnSe)_13_ magic‐sized clusters in terms of their energetic position and shape, indicating the successful formation of the host (ZnSe)_13_ magic‐sized clusters. Notably, the synthesized magic‐sized clusters exhibit additional transitions at the low energy range of 650–750 nm (inset of Figure 3b), which is attributed to the ^4^A_2_–^4^T_1_(P) ligand field transition of Co^2+^ ions. The presence of the ^4^A_2_–^4^T_1_(P) ligand field transition demonstrates that Co^2+^ ions are substitutionally doped within the tetrahedral coordination of the host magic‐sized clusters.^[^ ^65^ ^]^ However, the synthesized quantum nanoribbons do not exhibit the ^4^A_2_–^4^T_1_(P) ligand field transition, suggesting that Co dopants are not in the tetrahedral sites of the quantum nanoribbons. Time‐dependent evolution of the absorption spectra during the formation of quantum nanoribbons from Co‐doped (ZnSe)_13_ magic‐sized clusters show that the internal crystal field transition disappeared during the transformation of the magic‐sized clusters into the quantum nanoribbons (Figure 3c,d). This further confirms that the Co dopants, which are originally located in the tetrahedral sites of (ZnSe)_13_ magic‐sized clusters, escape from the substitutional doping sites.

To identify the spatial distribution of Co dopants in the synthesized quantum nanoribbons, we employed EDS analysis (Figure 3e–i). The EDS mapping images and the corresponding EDS spectrum (Figure S22, Supporting Information) reveal that Co dopants remain in the synthesized ZnSe quantum nanoribbons, although they do not exist in the tetrahedral lattice sites. The presence of Co element is further confirmed by inductively coupled plasma optical emission spectrometer (ICP‐OES) elemental analysis (Figure S23 and S24, Supporting Information). It is worth noting that these elemental analyses were performed on the samples that had undergone rigorous purification with eight cycles of washing processes. Consequently, we can conclude that Co dopants are located at interstitial (or surface) sites in the ZnSe quantum nanoribbons, rather than being simply physically adsorbed. In this study, the term “interstitial” also includes surface doping (excluding simple physical adsorption), as a significant portion of the atoms in the nanoribbons are exposed on the surface due to their ultrathin structure. Moreover, a similar trend was observed in CdSe, indicating that the suggested nucleation doping mechanism may be generally applicable to group II–VI materials, as demonstrated in Figure S25–S30, Supporting Information.

Based on our results, we propose that the Co^2+^ ions in (ZnSe)_13_ magic‐sized clusters escape from their substitutional doping sites during the crystallization of ZnSe quantum nanoribbons. In contrast, substitutional doping of Mn dopants is maintained throughout the synthesis process. These findings suggest that the doping type can be controlled by the elemental species of dopants used in the synthesis process. We hypothesize that Co dopants do not remain substitutional in the final nanocrystals due to the relatively weak ionic bonding of Co—Se compared to Zn—Se and Mn—Se.^[^ ^66^ ^]^ This weaker bonding makes the tetrahedral coordination of Co—Se unstable in the 2D quantum nanoribbons, which experience significant lattice strain (e.g., the *d*‐spacing of the ($1\bar{1}00$) plane of ZnSe nanoribbons is 3.355 Å, which is considerably smaller than 3.461 Å observed in bulk ZnSe crystals).^[^ ^67^, ^68^ ^]^ Additionally, the ligand field theory suggests that Co^2+^ ions have a preference for octahedral coordination rather than the tetrahedral coordination found at substitutional doping sites in ZnSe systems.^[^ ^69^ ^]^ This preference likely further destabilizes Co dopants in tetrahedral sites, resulting in their relocation during the crystallization process.

**Figure 4:**
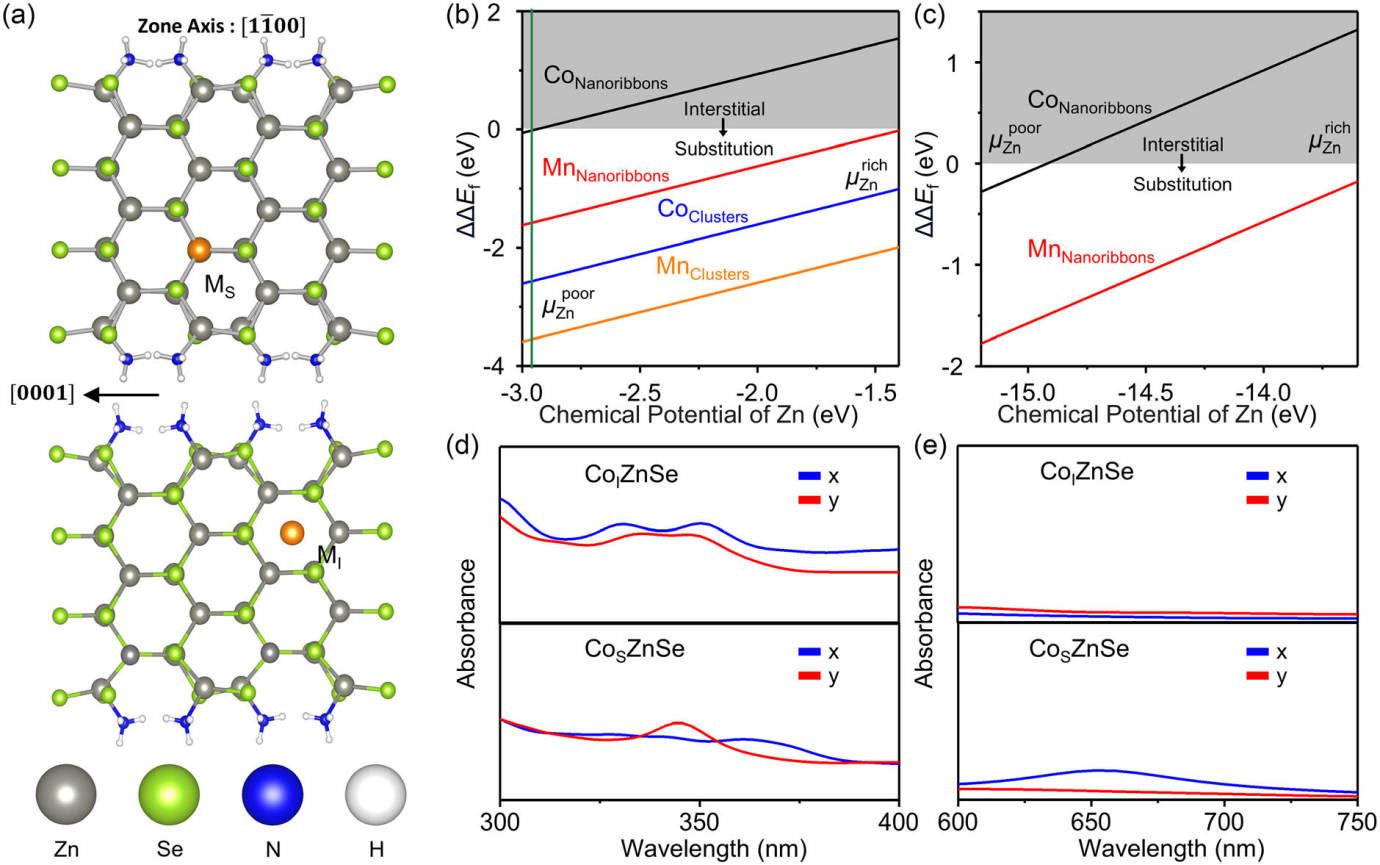
Computational insights into the thermodynamic stability of ZnSe quantum nanoribbons with substitutional and interstitial doping. a) A schematic depiction of the nanoribbon structure with substitutional (*M*_S_) and interstitial (*M*_I_) dopants. The gray, light green, blue, white, and orange balls represent the Zn, Se, N, H, and dopants, respectively. Calculated relative formation energy $\text{ΔΔ}E_{f}$ for substitutional versus interstitial doping as a function of the chemical potential of Zn using b) PBE and c) SCAN functional. The vertical green line indicates the chemical potential of Zn^2+^(aq). The calculated absorption spectra of interstitial Co‐doped ZnSe (Co_I_ZnSe) nanoribbons and substitutional Co‐doped ZnSe (Co_s_ZnSe) nanoribbons at d) short wavelength and e) long wavelength range.

To enhance our comprehension of the doping process, we performed extensive spin‐polarized DFT calculations (**Figure** **4**a). The objective of these calculations is to acquire a deeper understanding of the stability of dopants within quantum nanoribbons and magic‐sized clusters (see Supporting Methods in the Supporting Information for details). We first explored substitutional and interstitial doping into ZnSe quantum nanoribbons with functionalized surfaces, using ammonia as surface ligands. The most stable sites for substitutional and interstitial doping of ZnSe quantum nanoribbons found after rigorous sampling of doping sites are depicted in Figure S31–S34, Supporting Information.

To study in more detail the relative stability of interstitial and substitutional doping as a function of reference potential, we evaluated the relative formation energy of these two doping events (Figure 4b,c). We define this difference as

(1) $$\Delta \Delta E_{f}\left[D\right]=\Delta E_{f}\left(S\right)-\;\Delta E_{f}\left(I\right)\;+n_{\text{Zn}}\times \mu_{\text{Zn}}$$

where *E*_f_(*S/I*) are the formation energies of substitutional/interstitial dopants as defined in the Supporting Information, and *μ* depicts the chemical potentials of the metals (the DFT energy). The Zn chemical potential is considered as a variable from metal‐rich (pure metals) to poor (metal selenides) conditions. From this analysis, we find that in the quantum nanoribbons, Co doping prefers the interstitial sites while Mn doping prefers the substitutional sites. For the magic‐sized clusters, both dopants prefer the substitutional sites. These results are fully in line with the experimental observations, providing thermodynamic explanations. All the calculated formation energies are summarized in Table S2–S4, Supporting Information. The observed stabilization of the formation energy of ZnSe quantum nanoribbons with the interstitial Co doping is also attributed to the trend of charge transfer, where ≈0.20 electron charge is transferred from the adjacent Zn atoms to the dopants. Conversely, when Co dopants are introduced at the substitutional site, it contributes ≈0.32 electrons to the adjacent selenium atoms.

To connect these trends with the experimentally measured absorption spectrum, we simulated the band structure from standard hybrid functional DFT^[^ ^70^ ^]^ for Co dopants at the substitutional and interstitial sites of the quantum nanoribbons, respectively (Figure 4d,e, absorbance along the in‐plane direction is shown). The optical properties are computed by neglecting local field effects from the frequency‐dependent dielectric matrix^[^ ^71^, ^72^ ^]^ (for computational details, see Supporting Methods in the Supporting Information for details). Interestingly, the simulated absorption spectra show almost quantitative agreement with the experimental measurements. Both simulated spectra (either interstitial or substitutional doping) display two absorption peaks at the short wavelength region (300–400 nm), closely aligned with the experimentally observed heavy and light hole‐excitonic transitions (Figure 3a). Furthermore, the simulated spectrum of quantum nanoribbons with interstitial Co doping does not exhibit additional transitions at the wavelength of 650–750 nm, while that of quantum nanoribbons with substitutional Co doping shows the additional transitions at this range, corresponding to the Co ^4^A_2_–^4^T_1_(P) ligand field transition. These findings further support the interstitial Co doping of the final 2D nanocrystals observed in the experiments.

## 3 Conclusions

**Figure 5:**
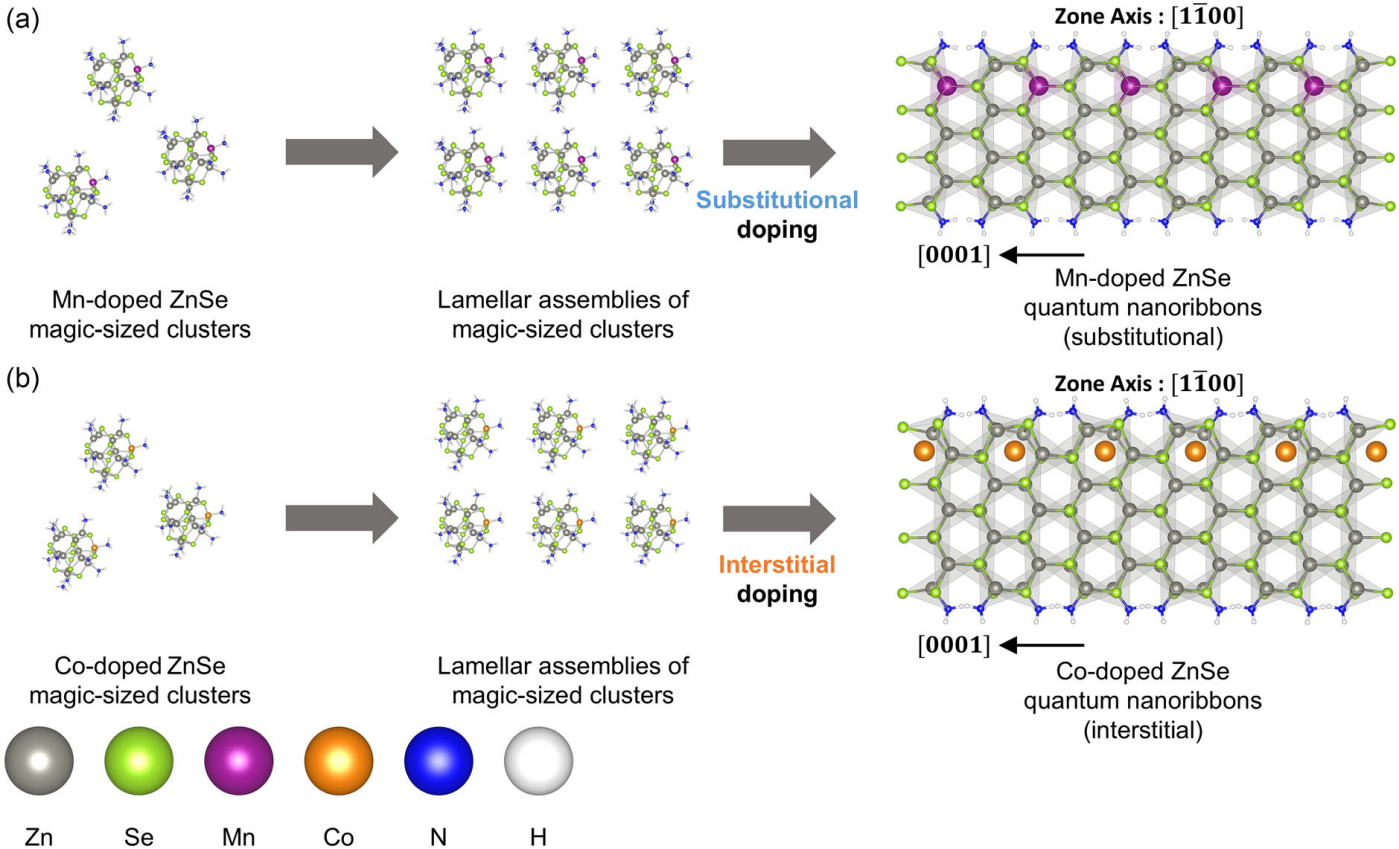
Schematic illustration showing the nucleation‐controlled doping process. a) Doping mechanism of Mn‐doped and b) Co‐doped ZnSe quantum nanoribbons. Gray, light green, purple, orange, blue, white, and green balls represent the Zn, Se, Mn, Co, N, H, and Cl atoms, respectively. The dopants are represented in a larger size to highlight their presence and distinguish them from the host atoms.

In summary, we have elucidated the nucleation‐controlled doping process via magic‐sized cluster intermediates, resulting in the synthesis of 2D ZnSe quantum nanoribbons with different doping types (**Figure** **5**). Our study presents a reliable synthesis method for doped 2D ZnSe quantum nanoribbons and validates the doping process through a comprehensive array of analytical techniques, including TEM, optical spectroscopy, EXAFS, and in situ SAXS analyses. We have demonstrated that the choice of dopants significantly influences the doping pathway of nanocrystals. Mn doping maintains the substitutional doping state throughout the synthesis process (Figure 5a), whereas Co dopants migrate from substitutional sites in magic‐sized clusters to alternative sites, such as interstitial sites, in the final nanocrystals (Figure 5b). Furthermore, DFT calculations have provided critical insights by revealing that these two dopants in ZnSe quantum nanoribbons exhibit distinct thermodynamic preferences for doping sites, offering strong support for the proposed doping mechanism. Overall, our study provides valuable insights into the nucleation‐controlled doping process, significantly advancing our understanding of the synthesis of doped semiconductor nanocrystals. We envision that the elucidated doping process will pave the way for successful doping of various semiconductor nanocrystals, enabling prediction of chemical pathways and final products of the synthesis process. This will open up exciting possibilities for developing advanced nanocrystals with tailored properties for numerous applications.

## 4 Experimental Section

4.1

4.1.1

##### Materials

Zinc chloride (ZnCl_2_, 99.999%), cadmium chloride (CdCl_2_, 99.99%), manganese chloride (MnCl_2_, 99.99%), cobalt chloride (CoCl_2_, 99.9%), selenium (Se, 99.99%), *n*‐octylamine (99%), and trioctylphosphine (TOP, 90%) were purchased from Sigma–Aldrich. Anhydrous ethyl alcohol (EtOH, 99.9%) was purchased from Samchun Chemicals. CO gas (99.999%) was purchased from Sumitomo Seika Chemicals.

##### Synthesis of 2D ZnSe Quantum Nanoribbons

2D Mn‐doped ZnSe quantum nanoribbons were synthesized by the reaction between the mixture of cation precursors containing ZnCl_2_–octylamine and MnCl_2_–octylamine complexes and *n*‐octylammonium selenocarbamate in *n*‐octylamine. The cation precursor solution was prepared by heating 10.0 mL of *n*‐octylamine containing 1.5 mmol ZnCl_2_ and a controlled amount of MnCl_2_ at 120 °C for 2 h. The anion precursor was synthesized by bubbling CO gas into 5.0 mL *n*‐octylamine containing 4.5 mmol of selenium for 1.5 h and then injected into the metal halide–octylamine solution. The mixture was kept at the room temperature for 1 h to promote the formation of ZnSe magic‐sized clusters. Then, the reaction mixture was heated to 150 °C and kept at this temperature for 16 h. The resulting quantum nanoribbons were subjected to purification by the centrifugation using ethanol containing several drops of TOP, followed by an additional centrifugation using pure ethanol. All procedures were conducted under an Ar environment using Schlenk techniques. For the synthesis of 2D Co‐doped ZnSe quantum nanoribbons, CoCl_2_ was used instead of MnCl_2_.

##### Synthesis of 2D CdSe Quantum Nanoribbons

Mn‐doped CdSe quantum nanoribbons were synthesized following a previously reported method.^[^ ^7^ ^]^ For the synthesis of Co‐doped CdSe quantum nanoribbons, the same method was employed, substituting MnCl_2_ with CoCl_2_.

##### Synthesis of ZnSe Magic‐Sized Clusters

The synthesis of Mn‐doped ZnSe clusters was carried out via reactions between the mixture of cation precursors consisting of ZnCl_2_–octylamine and MnCl_2_–octylamine complexes, and *n*‐octylammonium selenocarbamate. The cation precursor solution was prepared by heating 10.0 mL of *n*‐octylamine containing 1.5 mmol ZnCl_2_ and a controlled amount of MnCl_2_ at 120 °C for 2 h. The anion precursor was synthesized by bubbling CO gas into 5.0 mL *n*‐octylamine containing 4.5 mmol of selenium for 1.5 h and then injected into the metal halide–octylamine solution. After 24 h of the reaction, the magic‐sized clusters were purified by the centrifugation using ethanol containing several drops of TOP, followed by an additional centrifugation using pure ethanol. All procedures were conducted under an Ar environment using Schlenk techniques. For the synthesis of Co‐doped ZnSe clusters, CoCl_2_ was used instead of MnCl_2_. For the synthesis of undoped ZnSe clusters, neither CoCl_2_ nor MnCl_2_ was employed.

##### Material Characterization

The absorption spectra were acquired using a Cary 5000 spectrophotometer (Agilent), and the PL and PLE spectra were obtained using a Fluoromax‐4 (Horiba) spectrophotometer. For the analysis of time‐dependent optical spectra, 0.5 mL of the reaction mixture was collected and purified via the centrifugation. TEM images were obtained with a Tecnai G2 F20 TWIN TMP (Thermo Fisher Scientific) and a Titan Themis Z (Thermo Fisher Scientific). HAADF‐STEM and EDS analyses were carried out using a Titan Themis Z. ICP‐OES was carried out using an iCAP7400DUO (Thermo Fisher Scientific) instrument.

## Conflict of Interest

The authors declare no conflict of interest.

## Author Contributions

**Seunghyun Ji**: Data curation (lead); Formal analysis (lead); Investigation (lead); Methodology (lead); Visualization (lead); Writing—original draft (lead); Writing—review and editing (lead). **Hafiz Ghulam Abbas**: Conceptualization (lead); Data curation (lead); Formal analysis (lead); Investigation (lead); Methodology (lead); Validation (lead); Visualization (lead); Writing—original draft (lead); Writing—review and editing (lead). **Seo Young**: Conceptualization (lead); Data curation (lead); Formal analysis (lead); Investigation (lead); Methodology (lead); Validation (lead); Visualization (lead); Writing—original draft (lead); Writing—review and editing (lead). **Hyo Cheol Lee**: Data curation (supporting); Formal analysis (supporting); Investigation (supporting); Methodology (supporting); Validation (supporting). **Kyunghoon Lee**: Data curation (supporting); Formal analysis (supporting); Investigation (supporting); Methodology (supporting); Validation (supporting). **Shi Li**: Data curation (supporting); Formal analysis (supporting); Investigation (supporting); Validation (supporting). **Seungho Choe**: Data curation (supporting); Formal analysis (supporting); Investigation (supporting); Methodology (supporting). **Hyungju Ahn**: Data curation (supporting); Formal analysis (supporting); Investigation (supporting); Methodology (supporting). **Stefan Ringe**: Conceptualization (supporting); Data curation (lead); Formal analysis (lead); Funding acquisition (lead); Investigation (lead); Methodology (lead); Project administration (lead); Resources (lead); Supervision (lead); Validation (lead); Visualization (lead); Writing—review and editing (lead). **Jiwoong Yang**: Conceptualization (lead); Data curation (lead); Formal analysis (lead); Funding acquisition (lead); Investigation (lead); Methodology (lead); Project administration (lead); Resources (lead); Supervision (lead); Validation (lead); Visualization (supporting); Writing—original draft (lead); Writing—review and editing (lead). **Seunghyun Ji**, **Hafiz Ghulam Abbas**, and **Seo Young Kim** contributed equally to this work.

## Supporting information

Supplementary Material

## Data Availability

The data that support the findings of this study are available in the supplementary material of this article.

## References

[1] D. Mocatta , G. Cohen , J. Schattner , O. Millo , E. Rabani , U. Banin , Science 2011, 332, 77. 21454783 10.1126/science.1196321

[2] L. Wang , Z. Chen , G. Liang , Y. Li , R. Lai , T. Ding , K. Wu , Nat. Commun. 2019, 10, 4532. 31586066 10.1038/s41467-019-12558-y PMC6778069

[3] M. Shim , P. Guyot‐Sionnest , Nature 2000, 407, 981. 11069172 10.1038/35039577

[4] N. Grumbach , A. Rubin‐Brusilovski , G. I. Maikov , E. Tilchin , E. Lifshitz , J. Phys. Chem. C 2013, 117, 21021.

[5] P. Chakraborty , Y. Jin , C. J. Barrows , S. T. Dunham , D. R. Gamelin , J. Am. Chem. Soc. 2016, 138, 12885. 27593346 10.1021/jacs.6b05949

[6] Z. Chen , C. Zhao , X. Zhou , L. Xiao , Z. Li , Y. Zhang , Small Sci. 2023, 3, 2300086. 10.1002/smsc.202300086 PMC11936037 40212301

[7] J. H. Yu , X. Liu , K. E. Kweon , J. Joo , J. Park , K.‐T. Ko , D. W. Lee , S. Shen , K. Tivakornsasithorn , J. S. Son , J.‐H. Park , Y.‐W. Kim , G. S. Hwang , M. Dobrowolska , J. K. Furdyna , T. Hyeon , Nat. Mater. 2010, 9, 47. 19915554 10.1038/nmat2572

[8] A. Sahu , M. S. Kang , A. Kompch , C. Notthoff , A. W. Wills , D. Deng , M. Winterer , C. D. Frisbie , D. J. Norris , Nano Lett. 2012, 12, 2587. 22533700 10.1021/nl300880g

[9] C. A. Stowell , R. J. Wiacek , A. E. Saunders , B. A. Korgel , Nano Lett. 2003, 3, 1441.

[10] P. I. Archer , S. A. Santangelo , D. R. Gamelin , Nano Lett 2007, 7, 1037. 17378618 10.1021/nl0702362

[11] D. J. Norris , N. Yao , F. T. Charnock , T. A. Kennedy , Nano Lett. 2001, 1, 3.

[12] D. A. Schwartz , N. S. Norberg , Q. P. Nguyen , J. M. Parker , D. R. Gamelin , J. Am. Chem. Soc 2003, 125, 13205. 14570496 10.1021/ja036811v

[13] N. Pradhan , D. Goorskey , J. Thessing , X. Peng , J. Am. Chem. Soc. 2005, 127, 17586. 16351071 10.1021/ja055557z

[14] V. Proshchenko , Y. Dahnovsky , J. Phys. Chem. C 2014, 118, 28314. 10.1039/c3cp55314k 24634919

[15] Y. Yang , O. Chen , A. Angerhofer , Y. C. Cao , J. Am. Chem. Soc. 2008, 130, 15649. 18950179 10.1021/ja805736k

[16] C. B. Murray , D. J. Norris , M. G. Bawendi , J. Am. Chem. Soc. 1993, 115, 8706.

[17] M. R. Bergren , P. K. B. Palomaki , N. R. Neale , T. E. Furtak , M. C. Beard , ACS Nano 2016, 10, 2316. 26811876 10.1021/acsnano.5b07073

[18] R. Liu , R. D. Priestley , J. Mater. Chem. A 2016, 4, 6680.

[19] G. M. G. Khalaf , M. Li , J. Yan , X. Zhao , T. Ma , H.‐Y. Hsu , H. Song , Small Sci. 2023, 3, 2300062. 10.1002/smsc.202300062 PMC11935977 40213524

[20] D. H. Jara , S. J. Yoon , K. G. Stamplecoskie , P. V. Kamat , Chem. Mater 2014, 26, 7221.

[21] I. Moreels , K. Lambert , D. Smeets , D. De Muynck , T. Nollet , J. C. Martins , F. Vanhaecke , A. Vantomme , C. Delerue , G. Allan , Z. Hens , ACS Nano 2009, 3, 3023. 19780530 10.1021/nn900863a

[22] H. S. Yang , E. H. Suh , S. H. Noh , J. Jung , J. G. Oh , K. H. Lee , D. Lee , J. Jang , Chem. Eng. J. 2023, 454, 140331.

[23] P. Zhang , M. Jiao , Y. Li , X. Ding , K. J. McHugh , L. Jing , Small Sci. 2024, 4, 2300081. 10.1002/smsc.202300081 PMC11935123 40212631

[24] X. Peng , L. Manna , W. Yang , J. Wickham , E. Scher , A. Kadavanich , A. P. Alivisatos , Nature 2000, 404, 59. 10716439 10.1038/35003535

[25] S. Choo , H. W. Ban , D. H. Gu , H. Jeong , S. Jo , S. Baek , W. Jo , J. S. Son , Small 2019, 15, e1804426. 30624025 10.1002/smll.201804426

[26] H. Li , C. Cheng , Z. Yang , J. Wei , Nat. Commun. 2022, 13, 6466. 36309504 10.1038/s41467-022-34263-z PMC9617972

[27] F. Qi , K.‐J. Jeong , J. Gong , Z. Tang , Acc. Chem. Res. 2022, 55, 2425. 35977155 10.1021/acs.accounts.2c00202

[28] H. Ma , S. Kang , S. Lee , G. Park , Y. Bae , G. Park , J. Kim , S. Li , H. Baek , H. Kim , J.‐S. Yu , H. Lee , J. Park , J. Yang , ACS Nano 2023, 17, 13734. 37399231 10.1021/acsnano.3c03103

[29] D. J. Shin , H. Jang , D. Kim , J. Y. Woo , Y. K. Lee , W. K. Bae , J. Kim , Y.‐S. Park , D. C. Lee , Appl. Surf. Sci. 2023, 614, 156160.

[30] S. C. Erwin , L. Zu , M. I. Haftel , A. L. Efros , T. A. Kennedy , D. J. Norris , Nature 2005, 436, 91. 16001066 10.1038/nature03832

[31] D. J. Norris , A. L. Efros , S. C. Erwin , Science 2008, 319, 1776. 18369131 10.1126/science.1143802

[32] J. D. Bryan , D. R. Gamelin , Progress in Inorganic Chemistry, John Wiley & Sons, Inc, Hoboken, USA 2005.

[33] M.‐H. Du , S. C. Erwin , A. L. Efros , Nano Lett. 2008, 8, 2878. 18680387 10.1021/nl8016169

[34] B. B. Srivastava , S. Jana , N. Pradhan , J. Am. Chem. Soc. 2011, 133, 1007. 21186798 10.1021/ja1089809

[35] R. Buonsanti , D. J. Milliron , Chem. Mater. 2013, 25, 1305.

[36] J. Yang , F. Muckel , W. Baek , R. Fainblat , H. Chang , G. Bacher , T. Hyeon , J. Am. Chem. Soc. 2017, 139, 6761. 28481516 10.1021/jacs.7b02953

[37] W. Baek , M. S. Bootharaju , S. Lorenz , S. Lee , S. Stolte , R. Fainblat , G. Bacher , T. Hyeon , Adv. Funct. Mater. 2021, 31, 2107447.

[38] Y.‐H. Liu , F. Wang , Y. Wang , P. C. Gibbons , W. E. Buhro , J. Am. Chem. Soc. 2011, 133, 17005. 21905688 10.1021/ja206776g

[39] J. Yang , R. Fainblat , S. G. Kwon , F. Muckel , J. H. Yu , H. Terlinden , B. H. Kim , D. Iavarone , M. K. Choi , I. Y. Kim , I. Park , H.‐K. Hong , J. Lee , J. S. Son , Z. Lee , K. Kang , S.‐J. Hwang , G. Bacher , T. Hyeon , J. Am. Chem. Soc. 2015, 137, 12776. 26431472 10.1021/jacs.5b07888

[40] J. Yang , F. Muckel , B. K. Choi , S. Lorenz , I. Y. Kim , J. Ackermann , H. Chang , T. Czerney , V. S. Kale , S.‐J. Hwang , G. Bacher , T. Hyeon , Nano Lett. 2018, 18, 7350. 30265545 10.1021/acs.nanolett.8b03627

[41] T.‐E. Hsieh , T.‐W. Yang , C.‐Y. Hsieh , S.‐J. Huang , Y.‐Q. Yeh , C.‐H. Chen , E. Y. Li , Y.‐H. Liu , Chem. Mater. 2018, 30, 5468.

[42] M. S. Bootharaju , W. Baek , G. Deng , K. Singh , O. Voznyy , N. Zheng , T. Hyeon , Chem 2022, 8, 2978.

[43] L. He , C. Luan , N. Rowell , M. Zhang , X. Chen , K. Yu , Acc. Chem. Res. 2021, 54, 776. 33533599 10.1021/acs.accounts.0c00702

[44] L. Wang , J. Hui , J. Tang , N. Rowell , B. Zhang , T. Zhu , M. Zhang , X. Hao , H. Fan , J. Zeng , S. Han , K. Yu , Adv. Sci. 2018, 5, 1800632. 10.1002/advs.201800632 PMC6299716 30581693

[45] Y. Kwon , S. Kim , NPG Asia Mater. 2021, 13, 37.

[46] D. C. Gary , M. W. Terban , S. J. L. Billinge , B. M. Cossairt , Chem. Mater. 2015, 27, 1432.

[47] Y. Li , N. Rowell , C. Luan , M. Zhang , X. Chen , K. Yu , Adv. Mater. 2022, 34, 2107940. 10.1002/adma.202107940 35119147

[48] F. Muckel , S. Lorenz , J. Yang , T. A. Nugraha , E. Scalise , T. Hyeon , S. Wippermann , G. Bacher , Nat. Commun. 2020, 11, 4127. 32807786 10.1038/s41467-020-17563-0 PMC7431586

[49] M. Liu , K. Wang , L. Wang , S. Han , H. Fan , N. Rowell , J. A. Ripmeester , R. Renoud , F. Bian , J. Zeng , K. Yu , Nat. Commun. 2017, 8, 15467. 28580962 10.1038/ncomms15467 PMC5494182

[50] N. D. Loh , S. Sen , M. Bosman , S. F. Tan , J. Zhong , C. A. Nijhuis , P. Král , P. Matsudaira , U. Mirsaidov , Nat. Chem. 2017, 9, 77. 27995918 10.1038/nchem.2618

[51] S. Ithurria , B. Dubertret , J. Am. Chem. Soc. 2008, 130, 16504. 19554725 10.1021/ja807724e

[52] J. Joo , J. S. Son , S. G. Kwon , J. H. Yu , T. Hyeon , J. Am. Chem. Soc. 2006, 128, 5632. 16637619 10.1021/ja0601686

[53] F. Zhang , S. Wang , L. Wang , Q. Lin , H. Shen , W. Cao , C. Yang , H. Wang , L. Yu , Z. Du , J. Xue , L. S. Li , Nanoscale 2016, 8, 12182. 27251020 10.1039/c6nr02922a

[54] S. Ithurria , D. V. Talapin , J. Am. Chem. Soc. 2012, 134, 18585. 23061923 10.1021/ja308088d

[55] A. B. Pun , S. Mazzotti , A. S. Mule , D. J. Norris , Acc. Chem. Res. 2021, 54, 1545. 33660971 10.1021/acs.accounts.0c00859

[56] Y. Wang , Y. Zhou , Y. Zhang , W. E. Buhro , Inorg. Chem. 2015, 54, 1165. 25602285 10.1021/ic502637q

[57] P. D. Cunningham , I. Coropceanu , K. Mulloy , W. Cho , D. V. Talapin , ACS Nano 2020, 14, 3847. 32105062 10.1021/acsnano.9b09051

[58] M. Nasilowski , B. Mahler , E. Lhuillier , S. Ithurria , B. Dubertret , Chem. Rev. 2016, 116, 10934. 27434678 10.1021/acs.chemrev.6b00164

[59] B. T. Diroll , B. Guzelturk , H. Po , C. Dabard , N. Fu , L. Makke , E. Lhuillier , S. Ithurria , Chem. Rev. 2023, 123, 3543. 36724544 10.1021/acs.chemrev.2c00436

[60] L. Manna , L. W. Wang , R. Cingolani , A. P. Alivisatos , J. Phys. Chem. B 2005, 109, 6183. 16851684 10.1021/jp0445573

[61] H. C. Lee , M. S. Bootharaju , K. Lee , H. Chang , S. Y. Kim , E. Ahn , S. Li , B. H. Kim , H. Ahn , T. Hyeon , J. Yang , Adv. Sci. 2024, 11, 2307600. 10.1002/advs.202307600 PMC10853705 38072639

[62] R. Beaulac , P. I. Archer , X. Liu , S. Lee , G. M. Salley , M. Dobrowolska , J. K. Furdyna , D. R. Gamelin , Nano Lett. 2008, 8, 1197. 18331001 10.1021/nl080195p

[63] Y. Yang , O. Chen , A. Angerhofer , Y. C. Cao , J. Am. Chem. Soc. 2006, 128, 12428. 16984188 10.1021/ja064818h

[64] S. Acharya , D. D. Sarma , N. R. Jana , N. Pradhan , J. Phys. Chem. Lett. 2010, 1, 485.

[65] P. V. Radovanovic , D. R. Gamelin , J. Am. Chem. Soc. 2001, 123, 12207. 11734020 10.1021/ja0115215

[66] L. Pauling , The Nature of the Chemical Bond: An Introduction to Modern Structural Chemistry, Cornell University Press, Ithaca, NY 1960.

[67] J. S. Son , X.‐D. Wen , J. Joo , J. Chae , S.‐I. Baek , K. Park , J. H. Kim , K. An , J. H. Yu , S. G. Kwon , S.‐H. Choi , Z. Wang , Y.‐W. Kim , Y. Kuk , R. Hoffmann , T. Hyeon , Angew. Chem. Int. Ed. 2009, 48, 6861. 10.1002/anie.200902791 19688802

[68] H. Ma , D. Kim , S. I. Park , B. K. Choi , G. Park , H. Baek , H. Lee , H. Kim , J.‐S. Yu , W. C. Lee , J. Park , J. Yang , Adv. Sci. 2023, 10, 2205690. 10.1002/advs.202205690 PMC9982559 36638252

[69] G. L. Miessler , D. A. Tarr , Inorganic Chemistry, Prentice‐Hall, London, England 1991.

[70] J. Paier , M. Marsman , K. Hummer , G. Kresse , I. C. Gerber , J. G. Angyán , J. Chem. Phys. 2006, 124, 154709. 16674253 10.1063/1.2187006

[71] M. Gajdoš , K. Hummer , G. Kresse , J. Furthmüller , F. Bechstedt , Phys. Rev. B. 2006, 73, 045112.

[72] A. Piróth , J. Sólyom , Fundamentals of the Physics of Solids: Volume II: Electronic Properties, Springer, Berlin Heidelberg 2008.

