# Supplementary material for: Nucleation‐Controlled Doping of II–VI Semiconductor Nanocrystals Mediated by Magic‐Sized Clusters
Source: Small Sci. 2024 Nov 15;5(1):2400300. doi: 10.1002/smsc.202400300 (PMC11935185; doi:10.1002/smsc.202400300)
Supplement: Supplementary file 1 — Supplementary Material [file SMSC-5-2400300-s001.pdf]

Supporting Information

Nucleation-Controlled Doping of II-VI Semiconductor Nanocrystals Mediated by Magic-Sized Clusters

Seunghyun Ji,[1,†] Hafiz Ghulam Abbas,[2,†] Seo Young Kim,[1,†] Hyo Cheol Lee,[1] Kyunghoon Lee,[1] Shi Li,[1] Seungho Choe,[1,3] Hyungju Ahn,[4] Stefan Ringe,[2] & Jiwoong Yang[1,3]**

S. Ji, S. Y. Kim, H. C. Lee, K. Lee, S. Li, Prof. S. Choe, Prof. J. Yang
[1]Department of Energy Science and Engineering, Daegu Gyeongbuk Institute of Science and Technology (DGIST), Daegu 42988, Republic of Korea
*Email: jiwoongyang@dgist.ac.kr

Dr. H. G. Abbas, Prof. S. Ringe
[2]Department of Chemistry, Korea University, Seoul 02841 Republic of Korea
*Email : sringe@korea.ac.kr

Prof. S. Choe, Prof. J. Yang
[3]Energy Science and Engineering Research Center, Daegu Gyeongbuk Institute of Science and Technology (DGIST), Daegu 42988, Republic of Korea

Dr. H. Ahn
[4]Pohang Accelerator Laboratory, Pohang University of Science and Technology (POSTECH), Pohang 37673, Republic of Korea

[†]These authors contributed equally to this work.

Keywords: doping, semiconductor nanocrystals, magic-sized clusters, 2D nanocrystals, nucleation-controlled doping

Supporting Methods

X-ray absorption near edge spectroscopy (XANES) and extended X-ray absorption fine structure (EXAFS) analysis. XANES and EXAFS spectra were recorded at the PLS-II 10C beamline of the Pohang Accelerator Laboratory (PAL) in Korea. The energy calibration was performed by simultaneously measuring the spectra from reference samples. Dry powder samples were encapsulated within polyimide tape. Then, the spectra of each sample were measured with reference metal foils correspond to each atom (*e.g.*, Zn and Mn). Data were processed using Athena 0.9.26 software. For fitting the experimental data, Artemis 0.9.26 software was used and the amplitude reduction factor (S_0^2), the bond distances (R), and energy shifts (ΔE) were allowed to vary. Debye–Waller factors (σ^2) was set as fixed value. The hexagonal wurtzite crystal structure was considered for the analysis.

***In-situ* small-angle X-ray scattering (SAXS) analysis.** *In-situ* SAXS measurements were performed at the PLS-II 9A beamline of the Pohang Accelerator Laboratory (PAL) in Korea. Data acquisition utilized an MX170-HS (Rayonix) detector with the X-ray beam energy of 19.81 eV. The reaction mixture was prepared by combining the cation and anion precursor solutions following the experimental procedure for nanoribbon synthesis. A small quantity of the mixture was placed in a capillary, sealed with septum rubber, and positioned in a heating holder to capture the scattering pattern, as shown in Figure S15. The beam-exposure time and the time interval for each measurement was 5.0 s and 25 s, respectively. The distance between the sample and the detector was established at 2.0 m and the wave vector was ranging from 0.01 to ~0.6 Å^{-1}. One-dimensional scattering spectra were derived by azimuthal averaging of the two-dimensional scattering images. We recorded a scattering pattern from a capillary filled with only OcAm solvent, which served as the background. The background removal was carried out using the following equation,[S1]

$$I\ (q) = \frac{C(q)-C_d(q)}{T(\lambda)t} - \frac{C_b(q)-C_d(q)}{T_b(\lambda)t_b} \qquad \text{(S1)}$$

where $C(q)$ is the detected scattering intensity, $T(\lambda)$ is the absorption, $C_b(q)$ is the scattering of background, $C_d(q)$ is a dark current, and t is exposure time. $T_b(\lambda)$ and t_b are absorption and exposure times of background, respectively.

Laser desorption/ionization-mass spectrometry. Laser desorption/ionization-mass spectrometry was conducted with a Voyager-DE STR Biospectrometry Workstation (Applied Biosystems), operating in the negative mode. The desorption/ionization was accomplished using a nitrogen pulsed laser (3 ns pulses) with the laser intensity set to below ~40% of the maximum power to prevent fragmentation. The theoretical isotope distribution was determined using mMass 4.5 software.

Mott–Schottky (M–S) analysis. M–S analysis of quantum nanoribbons was conducted with an Autolab PGSTAT302N potentiostat (Metrohm) through a non-aqueous three-electrode system. Quantum nanoribbon-coated indium tin oxide (ITO) glass served as the working electrode, with a platinum wire as the counter electrode and a Ag/Ag^+ electrode immersed in an acetonitrile solution of 0.1 M tetra-*n*-butylammonium perchlorate (TBAP) and 0.01 M silver nitrate ($AgNO_3$) as the reference. The 0.1 M TBAP in acetonitrile was the electrolyte. To minimize the impact of oxygen, the electrolyte was saturated with argon gas for 30 min before testing. M–S tests varied in frequency from 100 kHz to 0.1 Hz, applying a signal amplitude of 5.0 mV. The carrier concentration (N) was calculated from the slopes of the M–S curves using following equation:

$$N\ (\mathrm{cm}^{-3}) = \left(\frac{2}{e\varepsilon\varepsilon_0}\right)\left[\frac{d(1/c^2)}{dV}\right]^{-1} \tag{S2}$$

where e is the elementary charge (1.6×10^{-19} C), ε is the relative dielectric constant of the semiconductor (8.976 for ZnSe quantum nanoribbons), ε_0 is the vacuum permittivity (8.8×10^{-14} F cm^{-1}), C (F cm^2) is the interfacial capacitance, and V (V) is the applied voltage.

Computational framework. Structural relaxations were performed using the Vienna ab initio simulation package (VASP).[S2,S3] The interactions between electrons and ions were described using the projector-augmented wave (PAW) method, involving a frozen-core all-electron calculation.[S3] Van der Waals interactions were considered by applying Grimme's correction to the Perdew (Burke) Ernzerhof method.[S4] For structure optimization, atoms were allowed to relax along the direction of the Hellmann-Feynman force, employing the conjugate gradient method with an energy cut-off of 400 eV until a strict convergence criterion of 0.001 eV$Å^{-1}$ was satisfied. The lattice constants were optimized using the PBE-D3 exchange-correlation

functional.[S5,S6] The electronic structure was computed using HSE06 and PBE-D3 exchange-correlation functionals.[S5-S7] We built the ZnSe quantum nanoribbons based on the optimized geometry of the bulk ZnSe wurtzite structure, placing it on the XY plane with the Z-axis aligned parallel to the c-axis. A substantial vacuum space of 36 Å was maintained along the Z-direction to prevent significant interactions between adjacent supercells. K-point sampling was performed using Γ-centered 4×4×1 k-points. The electronic and ionic contributions to the dielectric constant were computed using density functional perturbation theory. The total charge transfer was determined using a Bader charge population analysis.[S8,S9] The effective charge was calculated using the equation $Q_M = Z_M - q_{Bader}$, where Z_M represents the number of valence electrons of the dopants, and q_{Bader} represents the calculated Bader charge. We explicitly investigated the correction of the formation energy using a uniform scaling scheme for finite-size supercells, particularly for two-dimensional materials under the influence of a macroscopic field.[S10,S11]

In details, we constructed the hexagonal wurtzite bulk structure of ZnSe with the $p6_3mc$ space group and its unit cell comprising four atoms. We optimized its lattice parameters using the Perdew-Burke-Ernzerhof (PBE) functional,[S4] resulting in a, b = 4.0 Å and c = 6.57 Å. Next, we built ZnSe nanoribbons based on optimized geometry of its bulk structure. To accurately model the structure, we employed a 2×2 supercell model consisting of seven layers, comprising a total of 28 atoms positioned on the XY-plane. We maintained an ample vacuum space of 36 Å to avoid any significant interaction between the adjacent cells. During the optimization process of the structure, no constraints were employed on any of the layers. In addition, we used an ammonia (NH_3) molecules as surface ligands to functionalize the surface atoms. Our results showed a preference for NH_3 molecules to bind to the Zn site rather than the Se site. Furthermore, we explored two doping types in this study: (i) substitutional and (ii) interstitial doping. In the case of substitutional doping, we replaced cobalt (Co) or manganese (Mn) as the doping elements on the Zn sites of the structure as shown in Figure S31. We used the random alloy substitution method to generate numerous configurations considering the relaxed geometry of structure.

For all configurations, we adopted a two-step optimization process to determine the most stable structures resulting from the doping process. In the first step, we performed non-spin-polarized computations to obtain an optimized geometry. Building on this, we proceeded with spin-polarized calculations by considering the ferromagnetic coupling state, starting from the

geometry obtained in the previous step. This two-step approach ensured an accurate and reliable computational framework and allowed us to identify the ground state configurations for Co and Mn doping on the Zn sites of the quantum nanoribbons.

Throughout the optimization process, we utilized a Γ-centered 4×4×1 k-points sampling. Moving on to interstitial doping of Co and Mn on ZnSe nanoribbons, we investigated four potential sites for the interstitial dopants. These sites included the center of the hexagon, the Se–M–Se site, the Se–M–Zn site, and the Zn–M–Zn sites on the bottom sublayers as shown in Figures S32 and S33. Here, M represents the dopants (Co or Mn), respectively. Our findings showed that the hexagon site was the most stable binding site for Co dopant adsorption on this structure. Interestingly, after the second optimization, the Co atom slightly migrated towards the Se atoms of the bottom sublayer. Conversely, the most stable binding site for Mn dopant atom adsorption was where Mn bonded with three Se atoms two on the top sublayers and one on the bottom sublayers. Our calculation suggested that Co and Mn dopants preferred different doping sites. Additionally, we constructed ZnSe magic-sized clusters in various configurations, ensuring a 25 Å vacuum space along all directions to prevent interactions with neighboring cells. By replacing Zn atoms with Co or Mn, we introduced substitutional doping and all surface cations are passivated with NH_3 as surface ligands (Figure S34). Following our established methodology, all configurations were optimized using a Γ-centered k-points sampling method.

The optical properties were computed by neglecting local field effects from the frequency-dependent dielectric matrix:

$$\varepsilon(\omega) = \varepsilon_1(\omega) + \varepsilon_2(\omega) \qquad \text{(S3)}$$

where $\varepsilon_1(\omega)$ and $\varepsilon_2(\omega)$ are the real and imaginary parts of the dielectric function, respectively, and ω is the frequency of photon. In the context of one electron picture, the imaginary part of the dielectric function $\varepsilon_2(\omega)$ can be determined using the following equation:[S12]

$$\varepsilon_2(\omega) = \frac{4\pi^2 e^2}{\Omega} \lim_{q\rightarrow 0}\frac{1}{q^2}\sum_{c,v,k} 2\omega_k \delta(E_c - E_v - \omega)\,|< c|e.q|\,v >|^2 \qquad \text{(S4)}$$

where $< c|e.q|\,v >$ is the integrated optical transition from the valence states (v) to the conduction states (c), $\boldsymbol{e}$ is the polarization direction of the photon and $\boldsymbol{q}$ is the electron momentum operator. In this framework computing the real part of the dielectric function $\varepsilon_1(\omega)$,

and the integration over the electron wave vector **k** is achieved by summing over specific k-points and each k-point is associated with a corresponding weighting factor denoted as ω_k. The real part of the dielectric function $\varepsilon_1(\omega)$ can be determined using the Kramers-Kronig relation:

$$\varepsilon_1(\omega) = 1 + \frac{2}{\pi} P \int_0^\infty \frac{\varepsilon_2(\omega')\omega'}{\omega'^2 - \omega^2 + i\eta} d\omega' \quad \text{(S5)}$$

where P denotes the principle value and η is the complex shift parameter. The frequency-dependent absorption coefficient $\alpha(\omega)$ is computed from real $\varepsilon_1(\omega)$ and imaginary $\varepsilon_2(\omega)$ parts using the relation:[S13]

$$\alpha(\omega) = \frac{\sqrt{2}\omega}{c} \sqrt{\frac{|\varepsilon(\omega)| - \varepsilon_1(\omega)}{2}} \quad \text{(S6)}$$

$$|\varepsilon(\omega)| = \sqrt{\varepsilon_1^2 + \varepsilon_2^2} \quad \text{(S7)}$$

It should be noted that equation (Eq.) (S6) valid only for bulk structure and is not applicable for two dimensional (2D) systems. This limitation arises because the dielectric function becomes ambiguous and depends on the thickness of the vacuum layers when simulating 2D systems by employing a periodic stack of layers with a considerable interlayer distance (L).[S14,S15] The purpose of introducing ample vacuum along Z direction is to prevent artificial interactions between the adjacent images of the 2D sheet. To circumvent the issue related to thickness, we utilize the concept of optical conductivity to characterize the optical properties of 2D sheets. By relying on Maxwell's Eqs.[S16] the three-dimensional (3D) optical conductivity can be expressed as follows:

$$\sigma_{3D}(\omega) = [1 - \varepsilon(\omega)]\varepsilon_0 \omega \quad \text{(S8)}$$

where $\varepsilon(\omega)$ is the frequency-dependent complex dielectric function, ε_0 is the permittivity of vacuum and ω is the frequency of incident wave. The in-plane 2D optical conductivity is directly related to the corresponding $\sigma_{3D}(\omega)$ component as follows:[S14,S17]

$$\sigma_{2D}(\omega) = L\sigma_{3D}(\omega), \quad \text{(S9)}$$

where L represents the thickness of unit-cell along Z direction. So, the normalized absorbance A (ω) is independent of the light polarization for a freestanding 2D structure when normal incidence is assumed as follows:[S14,S15]

$$A = \frac{Re\tilde{\sigma}}{[1 + \tilde{\sigma}/2]^2} \tag{S10}$$

where

$$\tilde{\sigma}(\omega) = \frac{\tilde{\sigma}_{2D}(\omega)}{c\varepsilon_0} \tag{S11}$$

is the normalized conductivity and c is the speed of light. In the view of semiconducting and insulating 2D structures, the interband contribution is only taken into account when Eq. (S10) is valid, with the restriction A = 1. Typically, the absorbance (A) can be approximated by the real part (***Re***) of the optical conductivity $\tilde{\sigma}$ (ω), namely, Eq. (S12).

$$A(\omega) = \frac{Re\tilde{\sigma}_{2D}(\omega)}{c\varepsilon_0} \tag{S12}$$

The optical properties analysis was performed using the high-throughput tool vaspkit.[S18]

Formation energy framework. The stability of dopants (Co and Mn) in ZnSe on substitutional or interstitial sites was investigated by calculating their formation energies. The formation energy was determined using the following equation:

$$\Delta E_{\mathrm{f}}[D]^q = E_{\mathrm{tot}}(\mathrm{M@ZnSe})^q - E_{\mathrm{tot}}(\mathrm{ZnSe}) - \sum\nolimits_{\mathrm{i,\,m}}(n_{\mathrm{m}} \times \mu_{\mathrm{m}} - n_{\mathrm{i}} \times \mu_{\mathrm{i}}) + q \times (\varepsilon_{\mathrm{VBM}} + \epsilon_{\mathrm{f}}) + E_{\mathrm{corr}} \tag{S13}$$

where ΔE_{f} represents the dopant formation energy, E_{tot}(M@ZnSe) and E_{tot}(ZnSe) are the total ground state electronic energy of the doped and undoped systems, respectively. In Eq. (S13) number of atoms doped into the host material are represented by n_{i} , and guest metals (Co and Mn) atoms are denoted by n_{m}. The chemical potentials of the host μ_{i} and doped dopants are defined as μ_{m} and their source chemical potentials computed from their corresponding bulk structures. The term $\varepsilon_{\mathrm{VBM}}$ in Eq. (S13) signifies the valance band maximum eigenvalue obtained from a perfect unit cell based on the advanced HSE06 functional.[S7] Both (Co and Mn) dopants are denoted by the symbol M in Eq. (S13) and the Fermi level (ϵ_{f}) can vary within the material band gap.

To account for finite-size effects arising from the supercell approach, the energy term E_{corr} is introduced in Eq. (S13). This term corrects for these effects by utilizing the image charge correction and potential alignment correction methodology for charged dopants. In addition,

we used a charge correction scheme based on point charge through long-range Coulomb interaction proposed by Kumagai and Oba to accurately compute the formation energy of all bulk configurations.[S19] We opted to utilize this method instead of the dopant-induced potential alignment charge corrections method developed by the Van de Walle group, which can introduce significant errors for dopants in layered and 2D materials.[S20-S23] The main reason is that coulomb interactions in such materials are screened by a macroscopic scalar dielectric constant. The open-source Python package Spinney was used to calculate the finite size corrections.[S24]

The atomic chemical potential is related to the structure growth or doping conditions can be vary under the constraint determined by the phase equilibria. Considering the case of ZnSe as an example the relevant constraints are defined as Eq. (S14), Eq. (S15), and Eq. (S16):

$$\mu_{\mathrm{ZnSe(bulk)}} = \mu_{\mathrm{Zn}} + \mu_{\mathrm{Se}} \tag{S14}$$

$$\mu_{\mathrm{Zn}} \leq \mu_{\mathrm{Zn(metal)}} \tag{S15}$$

$$\mu_{\mathrm{Se}} \leq \frac{1}{3}\mu_{\mathrm{Se(bulk)}} \tag{S16}$$

where $\mu_{\mathrm{ZnSe(bulk)}}$, $\mu_{\mathrm{Zn(metal)}}$, and $\mu_{\mathrm{Se(bulk)}}$ are represents the chemical potential of bulk ZnSe, metal Zn, and bulk structure of Se.

The calculated ground state energies of these structures were used to calculate formation energies in Eq. (S13). Therefore, rich and poor limit conditions of metals (Zn, Mn and Co) atoms source chemical potentials are defined as follows:

$$\mu_{\mathrm{Zn}} = \mu_{\mathrm{ZnSe(bulk)}} - \frac{1}{3}\mu_{\mathrm{Se(bulk)}} \tag{S17}$$

$$\mu_{\mathrm{Co}} = \mu_{\mathrm{CoSe(bulk)}} - \mu_{\mathrm{Se(bulk)}} \tag{S18}$$

$$\mu_{\mathrm{Co}} \leq \mu_{\mathrm{Co(metal)}} \tag{S19}$$

$$\mu_{\mathrm{Mn}} = \mu_{\mathrm{MnSe(bulk)}} - \mu_{\mathrm{Se(bulk)}} \tag{S20}$$

$$\mu_{\mathrm{Mn}} \leq \mu_{\mathrm{Mn(metal)}} \tag{S21}$$

We computed the formation energies of metal-doped ZnSe quantum nanoribbons under two different (poor and rich) conditions. The formation energies, denoted as ϵ_{f}, show differences between the valence band maxima (VBM) and conduction band minima (CBM), except for

degenerate semiconductors where the Fermi level coincides with either the VB or CB. The position of the Fermi level relative to the VBM can be obtained as $\Delta\varepsilon_{\mathrm{f}}$.

$$\Delta\varepsilon_{\mathrm{f}} = \epsilon_{\mathrm{f}} - \varepsilon_{\mathrm{VBM}} \tag{S21}$$

Then, the variable range of $\Delta\varepsilon_{\mathrm{f}}$ becomes Eq. (S23).

$$0 \leq \Delta\varepsilon_{\mathrm{f}} \leq E_{\mathrm{g}} \tag{S23}$$

Depending on certain assumed chemical potential values, the formation energies are described as functions of $\Delta\varepsilon_{\mathrm{f}}$, we considered the charge state of dopants according to Eq. (S24).[S25]

$$\varepsilon\left(\frac{q}{q'}\right) = \frac{\Delta E_{\mathrm{f}}[D^{q}, \Delta\varepsilon_{\mathrm{f}}=0] - \Delta E_{\mathrm{f}}[D^{q'}, \Delta\varepsilon_{\mathrm{f}}=0]}{q-q'} \tag{S24}$$

where $\Delta E_{\mathrm{f}}[D^{q}, \Delta\varepsilon_{\mathrm{f}} = 0]$ denotes the formation energy of dopents for $\Delta\varepsilon_{\mathrm{f}} = 0$ at two different charge states is determined. Once the formation energies of the dopants were computed, equilibrium ϵ_{f} at a given temperature was obtained simultaneously with the equilibrium concentrations of charged dopants and carrier density.

If the contribution of vibrational entropy to Gibbs free energy and pressure can be considered negligible, concentration of impurities in dilute state (D^q) is given by Eq. (S25) as follows:[S22,S23,S26]

$$C[D^{q}] = N \times [D^{q}]\exp\left(-\frac{\Delta E_{\mathrm{f}}[D^{q}]}{k_{B}T}\right) \tag{S25}$$

where $N[D^{q}]$ is the number of sites per unit volume for dopants D^{q} tims its spin degeneracy, k_B is the Boltzmann constant, and T is absolute temperature. The hole density in the valence band (p) and electron density in the conduction band (n) are given as Eq. (S26) and Eq. (S27).[S10,S22,S23]

$$p = \int_{-\infty}^{\varepsilon_{\mathrm{VBM}}} D(\varepsilon)\frac{1}{\exp\left[{(\varepsilon_{\mathrm{f}}-\varepsilon)}/{k_{\mathrm{B}}T}\right] + 1}\, d\varepsilon \tag{S26}$$

$$n = \int_{\varepsilon_{\mathrm{CBM}}}^{\infty} D(\varepsilon)\frac{1}{\exp\left[{(\varepsilon-\varepsilon_{\mathrm{f}})}/{k_{\mathrm{B}}T}\right] + 1}\, d\varepsilon \tag{S27}$$

The electronic density of states (DOS, D(ε)) was calculated from the ideal crystal structure, considering that the presence of dopants does not significantly affect the DOS. The concentrations of dopants and carrier densities are subject to the charge neutrality condition,

ensuring that the total charge of the system remains balanced. The dopant concentrations and carrier densities are constrained by the charge neutrality condition as Eq. (S28).[S10,S22,S23]

$$p - n + \sum qC\,[D^q] = 0 \tag{S28}$$

Under the specified chemical potential conditions, the concentration of neutral dopants at a given temperature is determined solely by Eq. (S24). The self-consistent solution to Eq. (S26), Eq. (S27), and Eq. (S28) yields the concentrations of charged dopants, as well as the densities of carrier electrons and holes, and ϵ_{f} under thermal conditions. In this way, the interdependent variables were solved in a consistent manner, ensuring that the system properties are in equilibrium at the given temperature. We used an open-source Python package "*py-sc-fermi*" to calculate the concentration of dopants and charge carrier concentrations.[S12]

We used "*sxdefectalign2d*" tool to calculate corrections to the formation energy of localized charged dopants.[S10,S11] The dielectric constant ε calculated using density functional perturbation theory. The dielectric tensor components of the total system 6 along the C-axis (ε_{z}) and parallel to the slab ($\varepsilon_{\mathrm{xy}}$) can be written as Eq. (S29) and Eq. (S30).

$$\varepsilon_{xy} = \frac{\varepsilon s + v}{s + v} = 1 + (\varepsilon - 1)\frac{s}{c} \tag{S29}$$

$$\varepsilon_z^{\ -1} = \frac{\varepsilon^{-1} s + v}{s + v} = 1 - (\varepsilon - 1)\frac{s}{\varepsilon c} \tag{S30}$$

Here, the total height of the simulation cell is denoted by

$$c = s + v \tag{S31}$$

where *s* and *v* respectively represent the vacuum thickness and the center of the quantum nanoribbons slab. These equations can be reversed to obtain the model parameters from the dielectric tensor components. By utilizing Eq. (S29) and Eq. (S30), the overall dielectric constant of the slab can be deduced.

$$\varepsilon = \frac{\varepsilon_{xy} - 1}{1 - \varepsilon_z^{-1}} \tag{S32}$$

Supporting Figures

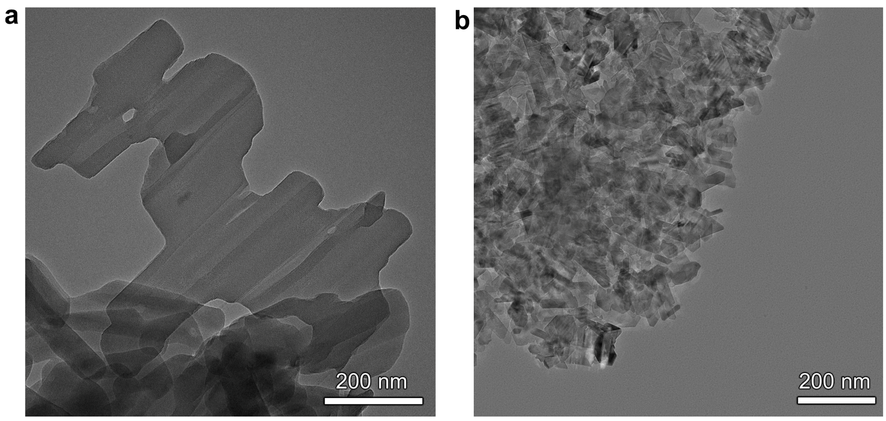

Figure S1. TEM images of samples from control experiments conducted at different heating temperatures: a) 70 °C and b) 170 °C. These control experiments followed the same procedure as the synthesis of Mn-doped ZnSe nanoribbons, with the exception of the heating temperature.

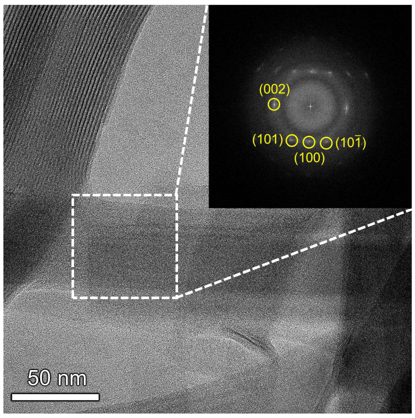

Figure S2. TEM image and the corresponding fast Fourier transform (FFT) pattern of Mn-doped ZnSe quantum nanoribbons (x_{Mn} = 12%).

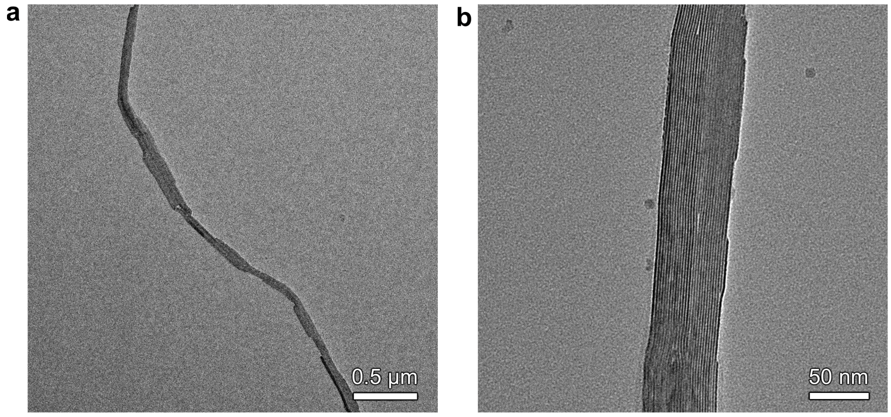

Figure S3. a) Low-resolution and b) high-resolution TEM images of undoped ZnSe quantum nanoribbons.

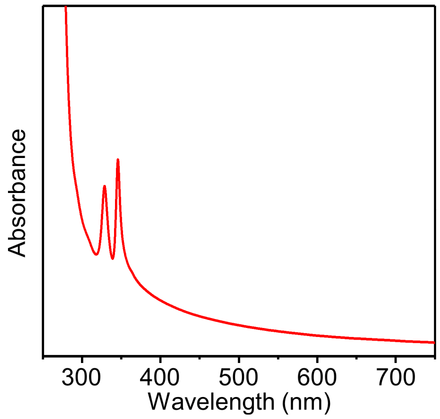

Figure S4. Absorption spectrum of undoped ZnSe quantum nanoribbons.

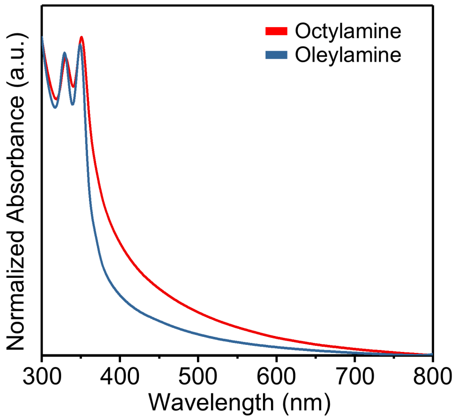

Figure S5. Absorption spectra of Mn-doped ZnSe quantum nanoribbons passivated with octylamine and oleylamine (x_{Mn} = 7%).

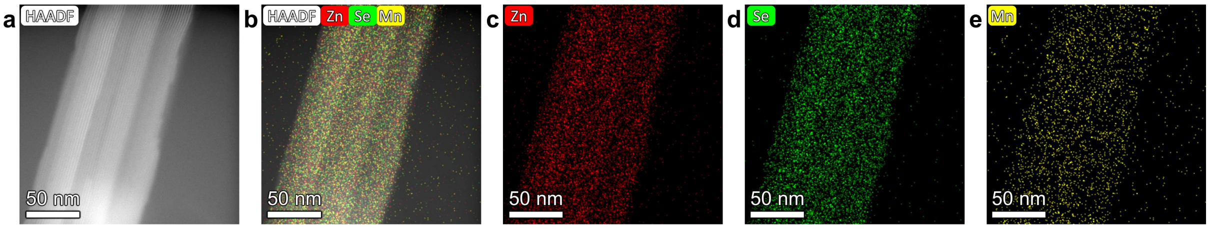

Figure S6. a) HAADF-STEM images and b-d) EDS mapping images of Mn-doped ZnSe quantum nanoribbons (x_{Mn} = 12%).

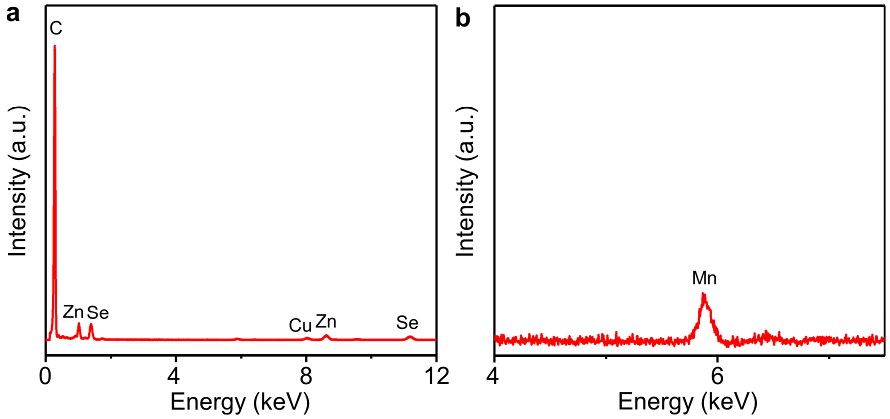

Figure S7. a) EDS spectrum and b) detailed spectrum of Mn-doped ZnSe quantum nanoribbons (x_{Mn} = 12%).

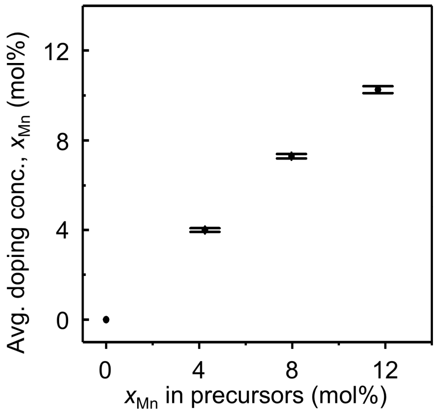

Figure S8. ICP-OES results of Mn-doped $(ZnSe)_{13}$ magic-sized clusters. The error bars represent standard deviations (n = 5).

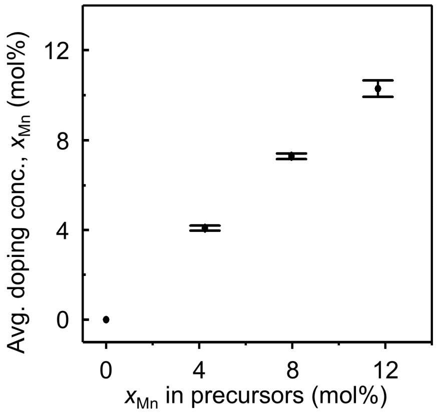

Figure S9. ICP-OES results of Mn-doped ZnSe quantum nanoribbons. The error bars represent standard deviations (n = 5).

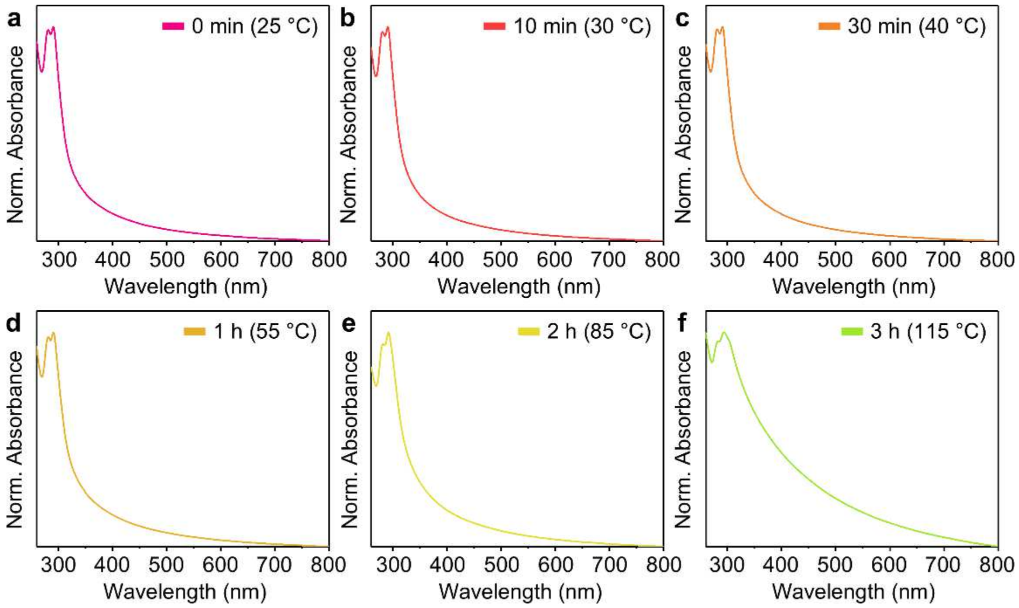

Figure S10. Normalized time-dependent absorption of a series of aliquots during the synthesis of Mn-doped ZnSe quantum nanoribbons (x_{Mn} = 7%) over a reaction time from 0 h to 3 h.

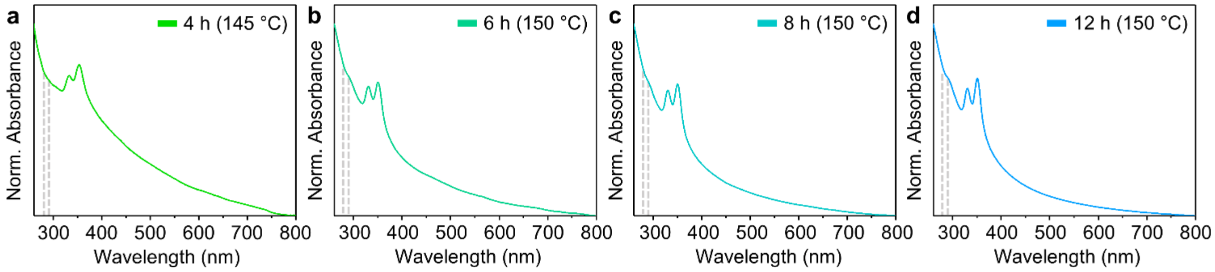

Figure S11. Normalized time-dependent absorption of a series of aliquots during the synthesis of Mn-doped ZnSe quantum nanoribbons (x_{Mn} = 7%) over a reaction time from 4 h to 12 h. The dashed lines indicate the positions of the characteristic transitions associated with $(ZnSe)_{13}$ magic-sized clusters.

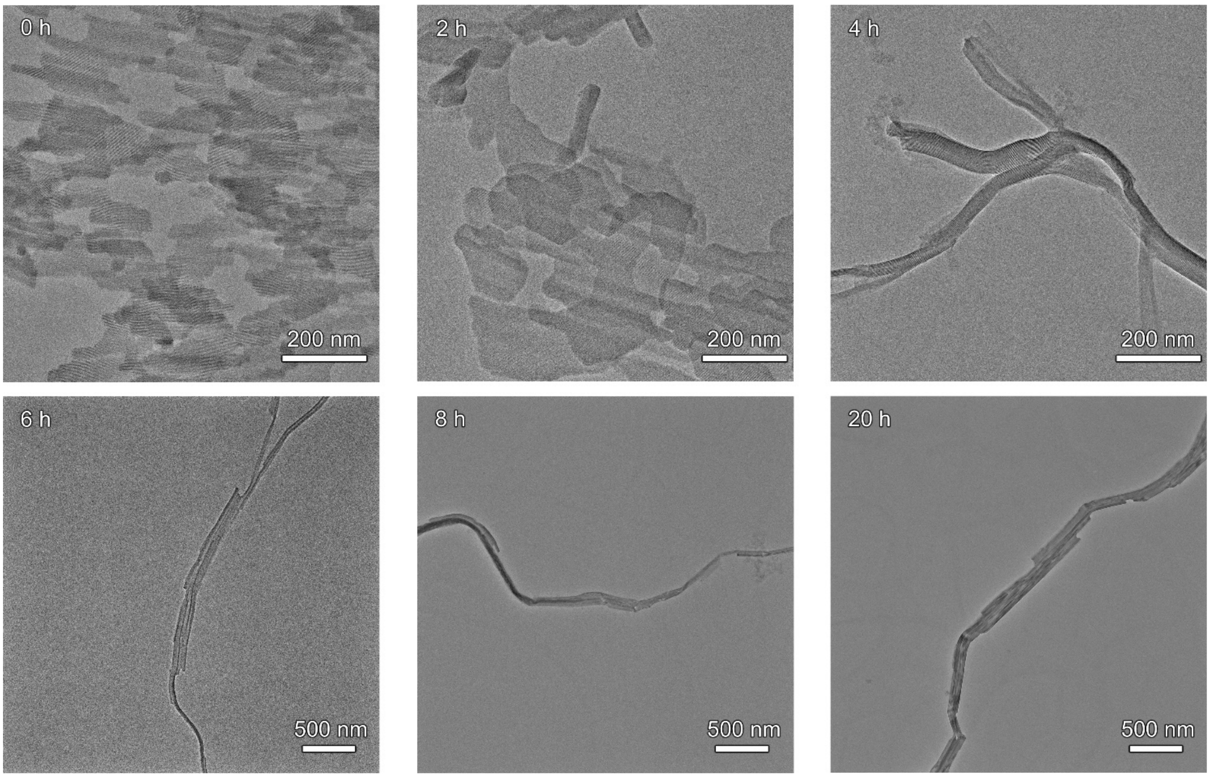

Figure S12. TEM images the aliquots samples during the synthesis of Mn-doped ZnSe quantum nanoribbons (x_{Mn} = 7%).

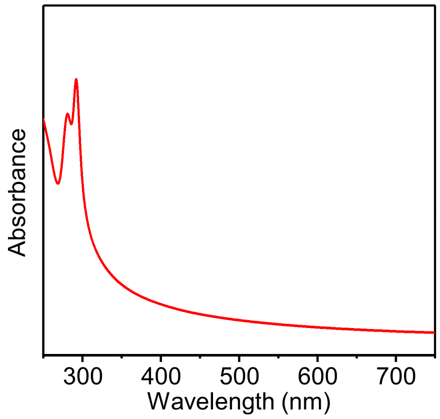

Figure S13. *Ex-situ* absorption spectrum of undoped $(ZnSe)_{13}$ magic-sized clusters.

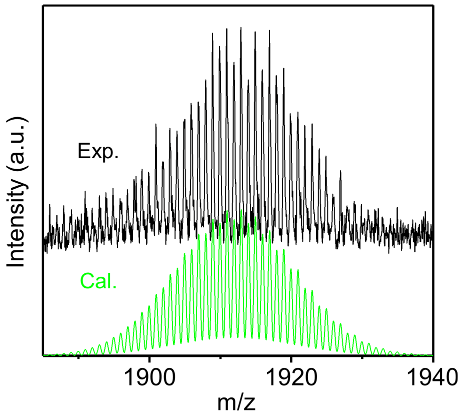

Figure S14. High-resolution laser desorption/ionization-mass spectrum of $(ZnSe)_{13}$ magic-sized clusters and their calculated isotopic distributions.

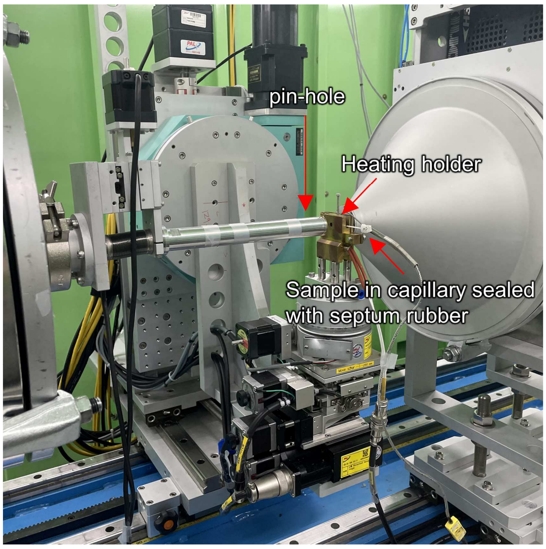

Figure S15. Photograph showing the experimental set-up for the *in-situ* SAXS measurement.

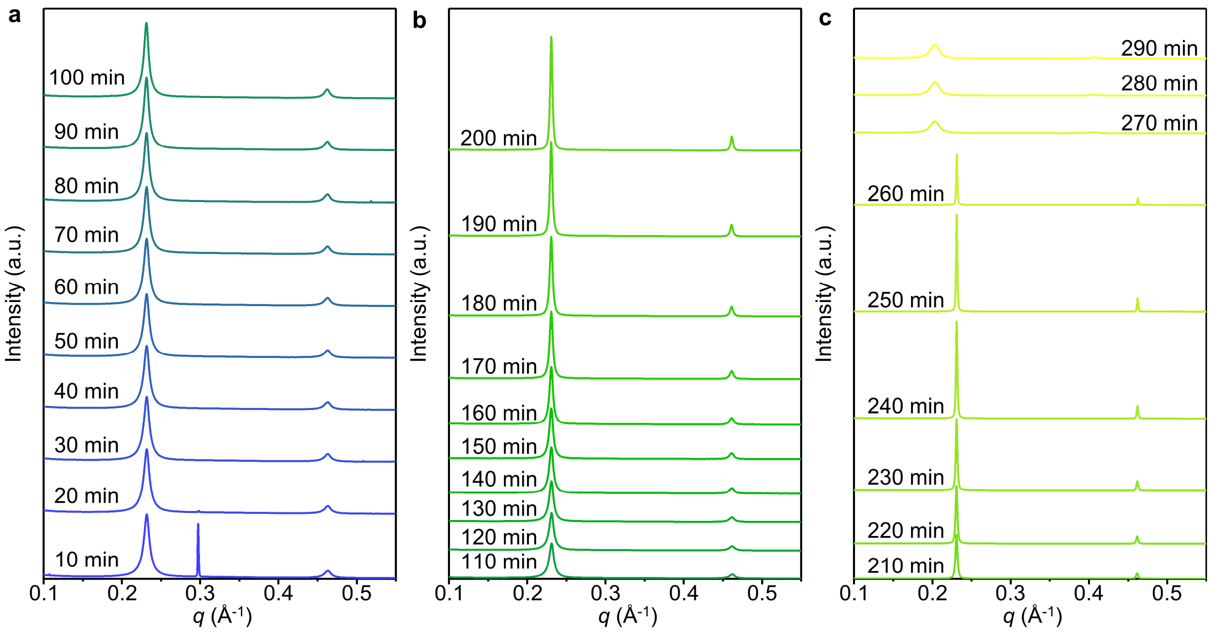

Figure S16. Representative *in-situ* SAXS patterns in Figure 2e, illustrating the evolution of Mn-doped ZnSe nanoribbons (x_{Mn} = 12%). The scattering patterns captured during a) 0–100 min, b) 110–200 min, and c) 210–290 min of the reaction.

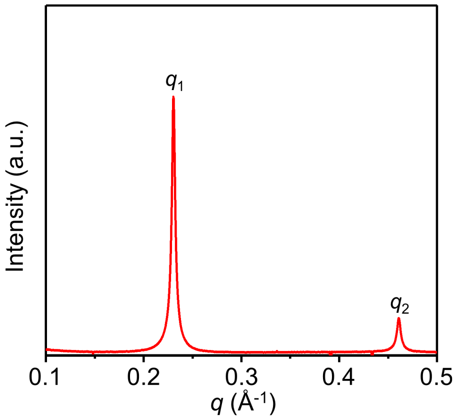

Figure S17. *Ex-situ* SAXS pattern of Mn-doped $(ZnSe)_{13}$ magic-sized clusters (x_{Mn} = 12%).

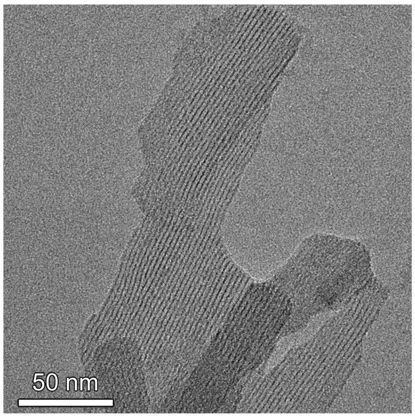

Figure S18. TEM image showing lamellar assemblies of Mn-doped $(ZnSe)_{13}$ magic-sized clusters (x_{Mn} = 7%). The inset image shows the enlarged images. The estimated center-to-center distance is ~2.71 nm, consistent with the value measured by SAXS data.

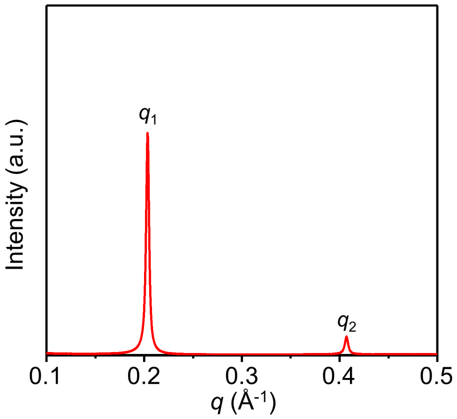

Figure S19. *Ex-situ* SAXS pattern of Mn-doped ZnSe quantum nanoribbons (x_{Mn} = 12%).

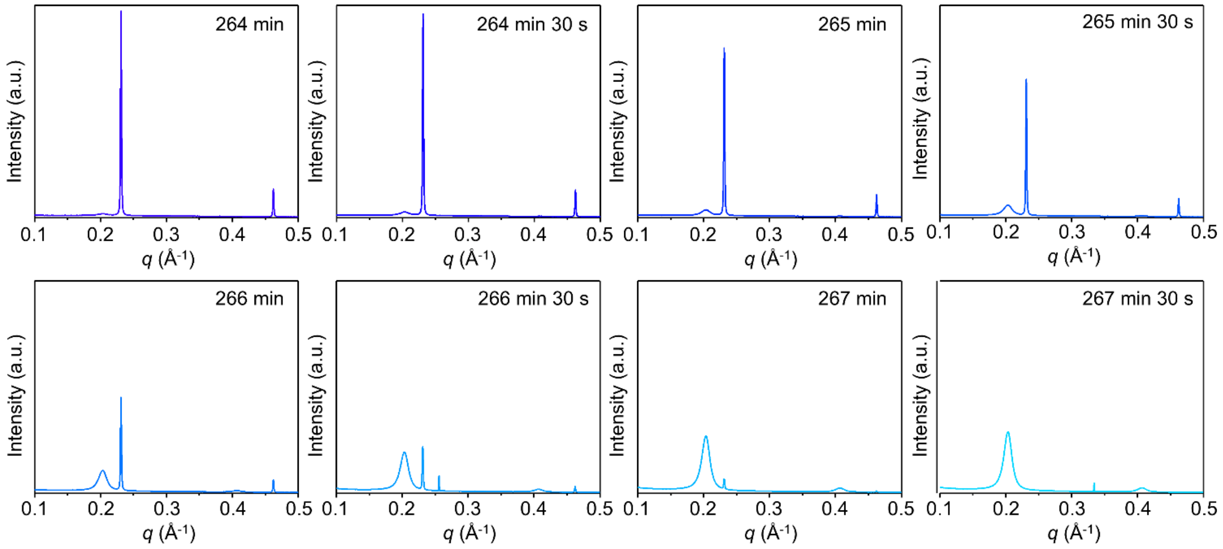

Figure S20. Representative *in-situ* SAXS patterns in Figure 2f, specifically focusing on the specific time intervals during the initial stages of Mn-doped ZnSe nanoribbon formation (x_{Mn} = 12%).

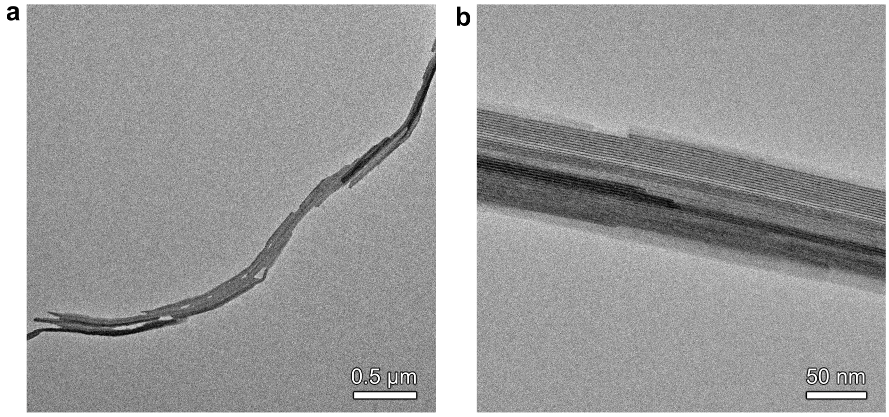

Figure S21. a) Low-resolution and b) high-resolution TEM images of Co-doped ZnSe quantum nanoribbons (x_{Co} = 11%).

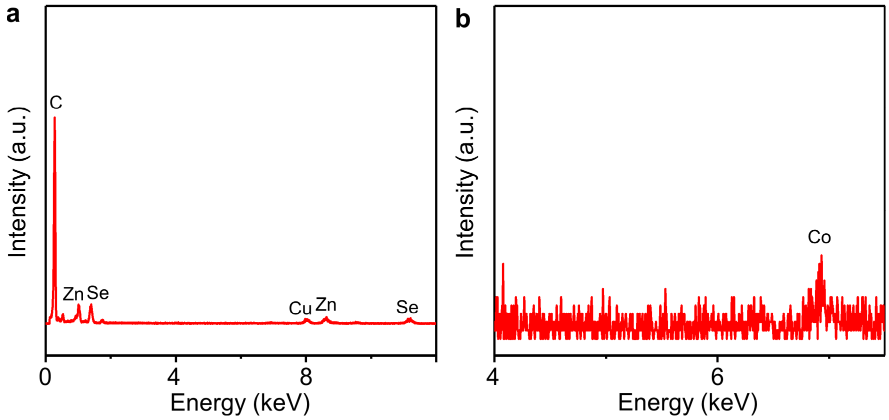

Figure S22. a) EDS spectrum and b) detailed spectrum of Co-doped ZnSe quantum nanoribbons (x_{Co} = 11%).

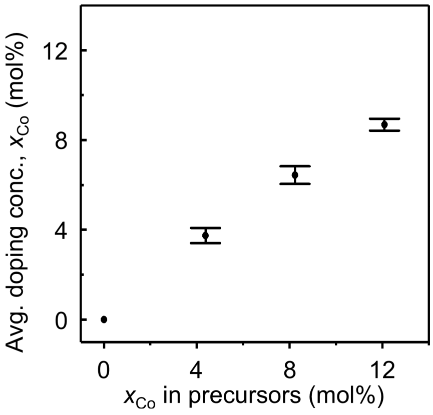

Figure S23. ICP-OES results of Co-doped $(ZnSe)_{13}$ magic-sized clusters. The error bars represent standard deviations (n=5).

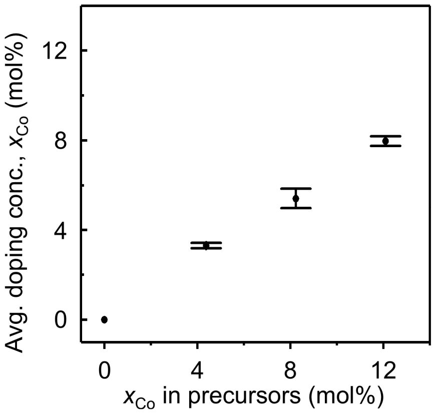

Figure S24. ICP-OES results of Co-doped ZnSe quantum nanoribbons. The error bars represent standard deviations (n=5).

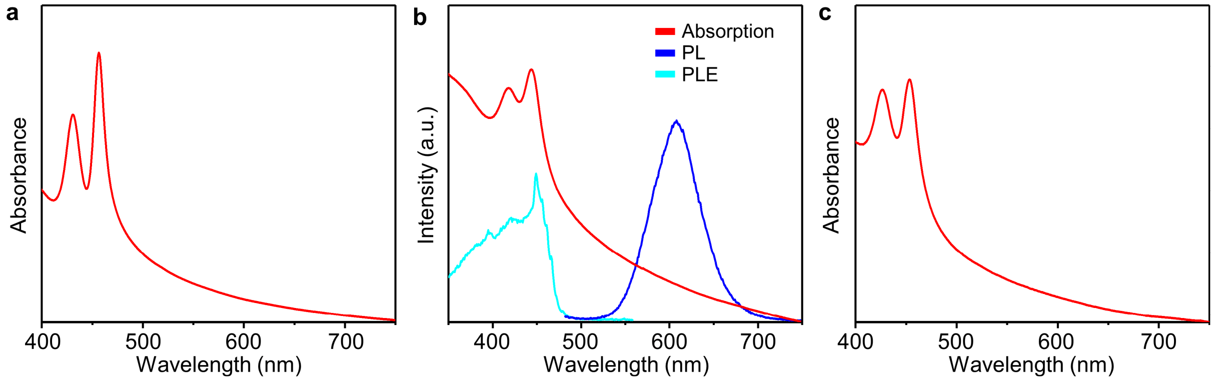

Figure S25. a) Absorption spectrum of undoped CdSe quantum nanoribbons. b) Absorption, PL, and PLE spectra of Mn-doped CdSe quantum nanoribbons (x_{Mn} = 7%). c) Absorption spectrum of Co-doped CdSe quantum nanoribbons (x_{Co} = 5%).

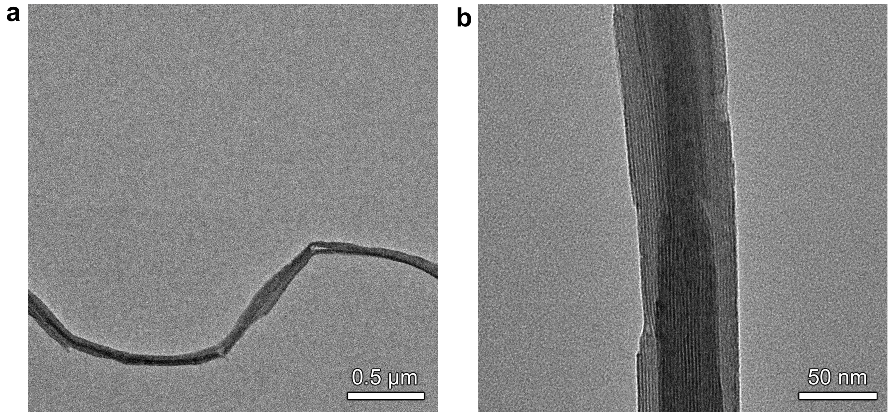

Figure S26. a) Low-resolution and b) high-resolution TEM images of undoped CdSe quantum nanoribbons.

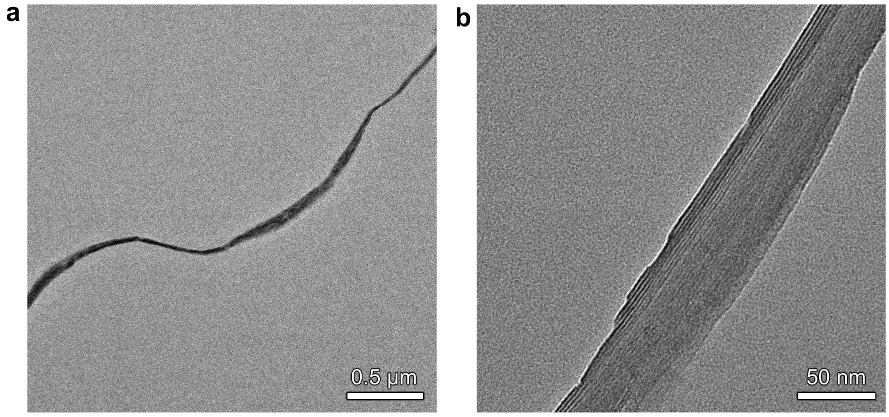

Figure S27. a) Low-resolution and b) high-resolution TEM images of Mn-doped CdSe quantum nanoribbons (x_{Mn} = 10%).

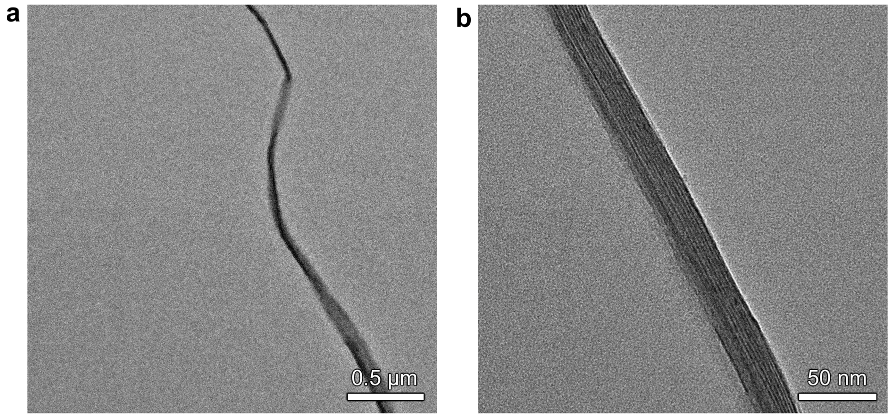

Figure S28. a) Low-resolution and b) high-resolution TEM images of Co-doped CdSe quantum nanoribbons (x_{Co} = 6%).

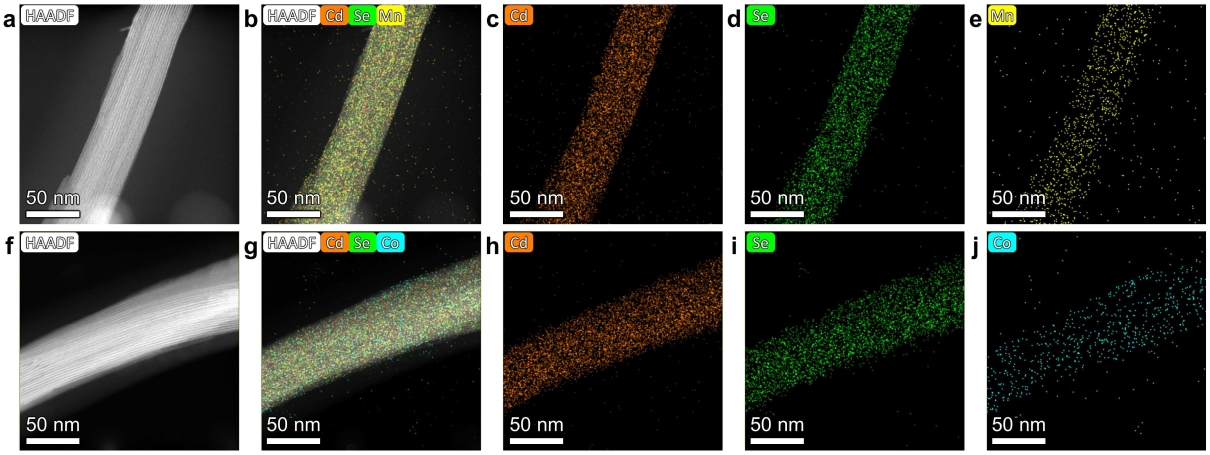

Figure S29. a) HAADF-STEM and b-e) EDS mapping images of Mn-doped CdSe quantum nanoribbons (x_{Mn} = 10%). f) HAADF-STEM and g-j) EDS mapping images of Co-doped CdSe quantum nanoribbons (x_{Co} = 6%).

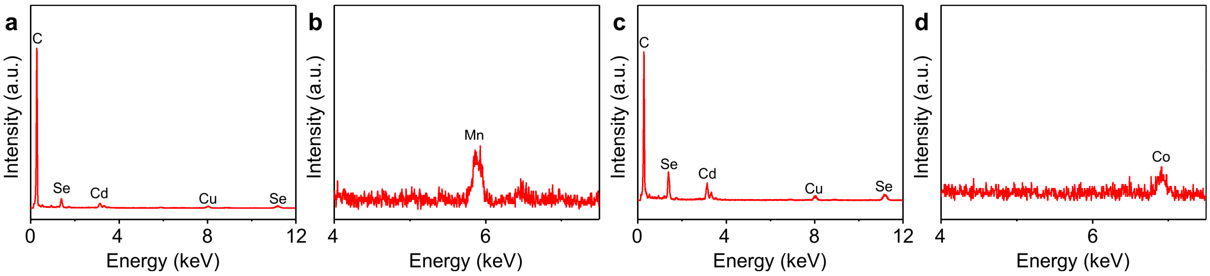

Figure S30. a) EDS spectrum of Mn-doped CdSe quantum nanoribbons (x_{Mn} = 10%) and b) enlarged spectrum. c) EDS spectrum of Co-doped CdSe quantum nanoribbons (x_{Co} = 6%) and d) enlarged spectrum.

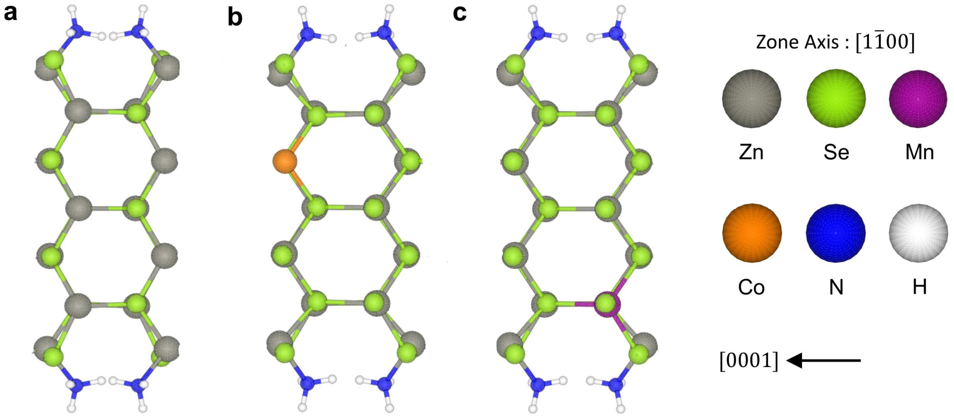

Figure S31. Chemical structures of the a) undoped ZnSe quantum nanoribbons and the most stable configurations of b) substitutional Co-doped ZnSe and, c) substitutional Mn-doped ZnSe quantum nanoribbons by replacing a single host atom in undoped ZnSe quantum nanoribbons. Here, we considered Mn or Co dopants to substitute Zn^{2+} ions in host materials. Surface Zn sites are passivated with NH_3 molecules. The gray, light green, purple, orange, blue, and white balls represent Zn, Se, Mn, Co, N, and H atoms, respectively.

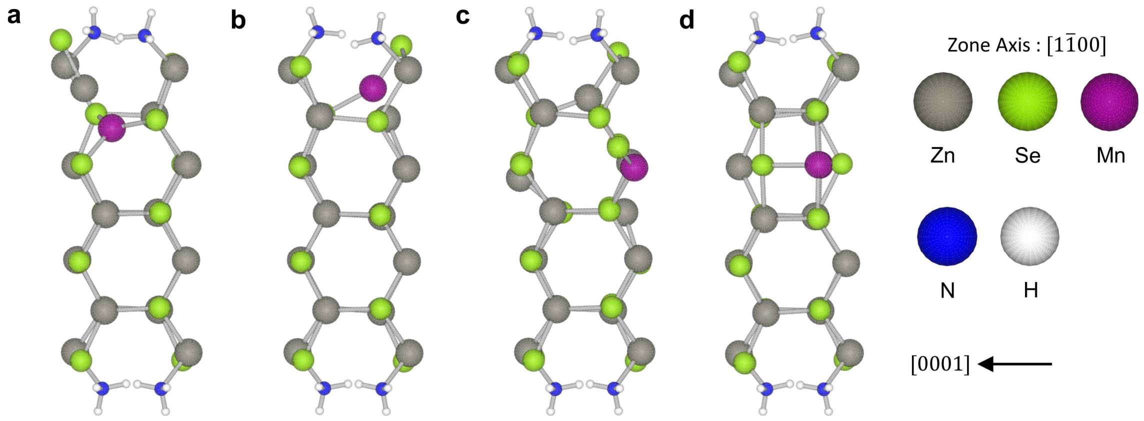

Figure S32. Chemical structures of the representative configurations of ZnSe quantum nanoribbons with an interstitial manganese (Mn_I) dopants. The gray, light green, purple, blue, and white balls represent Zn, Se, Mn, N, and H atoms, respectively.

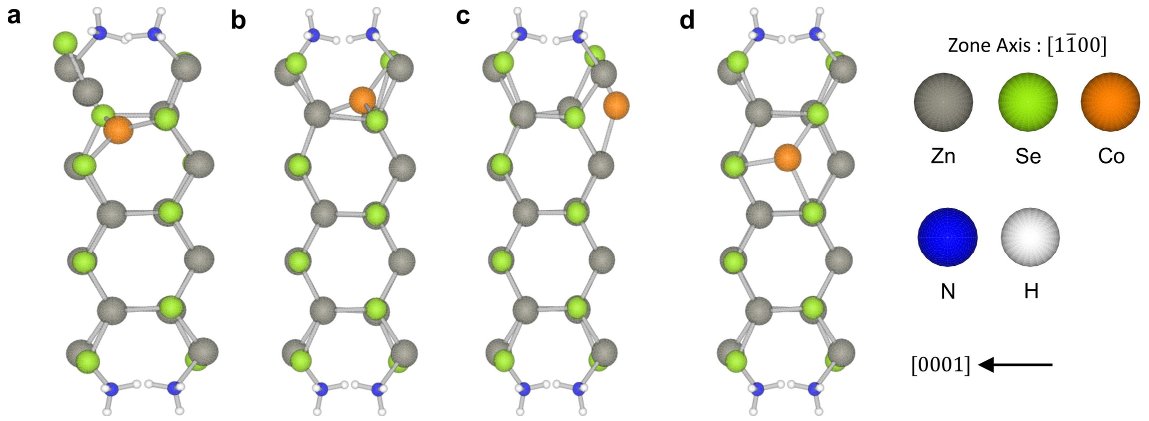

Figure S33. Chemical structures of the representative configurations of ZnSe quantum nanoribbons with interstitial cobalt (Co_I) dopants. The gray, light green, orange, blue, and white balls represent Zn, Se, Co, N, and H atoms, respectively.

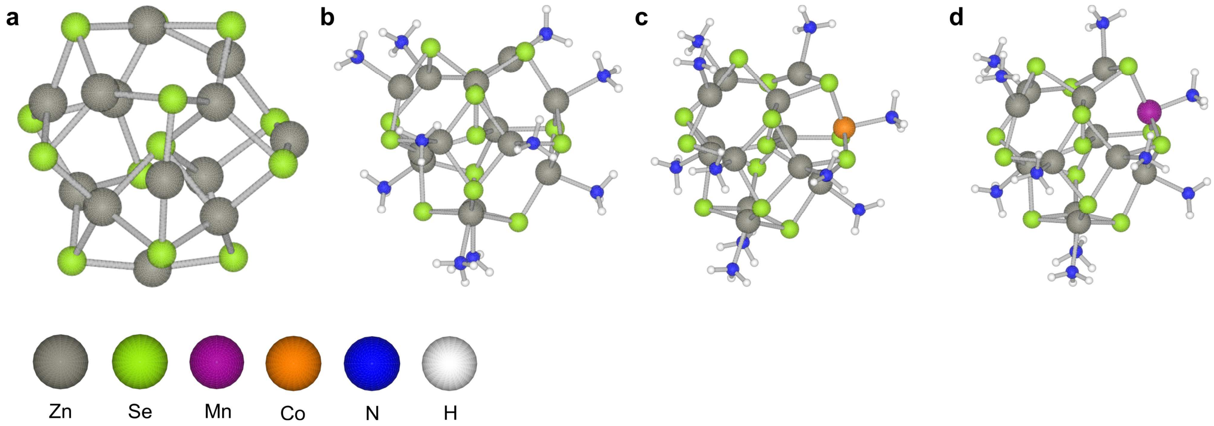

Figure S34. Chemical structures of a, b) undoped, c) substitutional Co-doped, and d) substitutional Mn-doped $(ZnSe)_{13}$ magic-sized clusters. Here, we consider Mn^{2+} or Co^{2+} ions as a dopant to substitute host Zn^{2+} ions in host materials. Surface Zn sites are passivated with NH_3 molecules. The gray, light green, purple, orange, blue, and white balls represent Zn, Se, Mn, Co, N, and H atoms, respectively.

Supporting Tables

Table S1. Results of EXAFS fitting analysis of Zn *K*-edge and Mn *K*-edge of undoped ZnSe quantum nanoribbons and Mn-doped ZnSe quantum nanoribbons (x_{Mn} = 12%). The fitting analysis was performed for ranges of 1.00–*R*–3.00 Å, 3.00–*k*–12.00 $Å^{-1}$.

Samples	Bond	Coordination number	Bond length (Å)	R-factor
Undoped ZnSe quantum nanoribbons	Zn–Se	4	2.440 ± 0.006	0.012
Mn-doped ZnSe quantum nanoribbons	Zn–Se	4	2.444 ± 0.005	0.010
Mn-doped ZnSe quantum nanoribbons	Mn–Se	4	2.531 ± 0.005	0.010

Table S2. The summary of calculation results for doped ZnSe nanoribbons using PBE functional.

**Samples**	**Configuration**	$\boldsymbol{\Delta E^{\mathrm{i}}_{\mathrm{f,NR}}}$ [a] **(eV)**	$\boldsymbol{\Delta E^{\mathrm{i}}_{\mathrm{f,bulk}}}$ [b] **(eV)**	$\boldsymbol{\Delta l^{\mathrm{NR}}_{\mathrm{M-Se}}}$ [c] **(Å)**	$\boldsymbol{\Delta l^{\mathrm{bulk}}_{\mathrm{M-Se}}}$ [d] **(Å)**	$\boldsymbol{q^{\mathrm{NR}}_{\mathrm{Bader}}}$ [e] **(e)**	$\boldsymbol{q^{\mathrm{bulk}}_{\mathrm{Bader}}}$ [e] **(e)**	$\boldsymbol{\mu^{\mathrm{NR}}_{\mathrm{B}}}$	$\boldsymbol{\mu^{\mathrm{bulk}}_{\mathrm{B}}}$
Co-doped ZnSe	Interstitial A (Figure S33a)	2.76	3.04	2.31	2.38	-0.09	-0.15	0.96	1.22
	Interstitial B (Figure S33b)	2.88	3.07	2.35	2.32	-0.11	0.06	0.96	1.24
	Interstitial C (Figure S33d)	3.04	3.22	2.39	2.35	-0.09	0.002	0.96	1.25
	Interstitial D (Figure S33c)	2.66	3.22	2.43	2.35	-0.11	0.003	0.96	1.22
	Substitutional (Figure S31b)	4.26	1.71	2.48	2.32	0.33	-0.06	2.26	2.11
Mn-doped ZnSe	Interstitial A (Figure S32a)	2.92	3.99	2.33	2.32	-0.70	-0.88	4.87	3.25
	Interstitial B (Figure S32b)	3.09	8.52	2.43	2.54	-0.55	-0.48	3.13	3.23
	Interstitial C (Figure S32c)	3.80	4.92	2.50	2.54	-0.55	-0.71	3.00	3.50
	Interstitial D (Figure S32d)	3.32	3.27	2.42	2.43	-0.67	-0.51	4.63	4.25
	Substitutional (Figure S31c)	2.86	0.95	2.59	2.33	-0.42	-0.53	5.0	5.0

[a]The formation energy of the dopants (M = Co or Mn) in ZnSe quantum nanoribbons, calculated within the level of PBE functional. The formation energy is defined as follows:

$$\Delta E_{\mathrm{f}} = [\, E_{\mathrm{tot}}(\mathrm{M@ZnSe–NH_3}) - E_{\mathrm{tot}}(\mathrm{ZnSe–NH_3}) - \textstyle\sum_{\mathrm{i,m}}(n_{\mathrm{m}}\,\mu_m - n_{\mathrm{i}}\mu_i)\,] \qquad \text{(S33)}$$

where E_{tot}(M@ZnSe–NH$_3$), E_{tot}(ZnSe–NH$_3$), and μ_m (Co and Mn), and μ_i (Zn) are represents the total ground state energy of the doped structure, undoped structure, and chemical potential of metals (Co, Mn, and Zn), respectively.

[b]The formation energy of the dopants (M = Co or Mn) in the bulk ZnSe structure, calculated within the level of PBE functional. The formation energy is defined as follows:

$$\varDelta E_{\mathrm{f}} = [\, E_{\mathrm{tot}}(\mathrm{M@ZnSe}) - E_{\mathrm{tot}}(\mathrm{ZnSe}) - \textstyle\sum_{\mathrm{i,m}}(n_{\mathrm{m}}\,\mu_m - n_{\mathrm{i}}\mu_i)\,] \qquad \text{(S34)}$$

where E_{tot}(M@ZnSe), E_{tot}(ZnSe), and μ_m(Co and Mn), and μ_i(Zn) are the total ground state energy of the doped structure, undoped structure, and chemical potential of metals (Co, Mn, and Zn), respectively.

[c]The average distance between the dopants (M = Co or Mn) and the neighboring Se atoms in the ZnSe quantum nanoribbons.

[d]The average distance between the dopants (M = Co or Mn) and the neighboring Se atoms in the bulk ZnSe structure.

[e]The total charge transfer from the dopants (M = Co or Mn) in ZnSe quantum nanoribbons and their corresponding bulk configurations, determined through Bader charge population analysis. The effective charge was calculated using as follows:

$$Q_M = Z_M - q_{Bader} \qquad \text{(S35)}$$

where Z_M represents the number of valence electrons of the dopants (M = Co or Mn), and q_{Bader} represents the calculated Bader charge. The positive and negative signs indicate the direction of charge transfer (loss or acceptance) from or towards the dopants.

Table S3. The summary of calculation results for doped ZnSe nanoribbons using SCAN functional.

Samples	Configuration	$\Delta E^{i}_{f,NR}$ [a] (eV)	$\Delta E^{i}_{f,bulk}$ [b] (eV)	Δl^{NR}_{M-Se} [c] (Å)	Δl^{bulk}_{M-Se} [d] (Å)	q^{NR}_{Bader} [e] (e)	q^{bulk}_{Bader} [e] (e)	μ^{NR}_{B}	μ^{bulk}_{B}
Co-doped ZnSe	Interstitial A (Figure S33a)	-0.53	2.95	2.31	2.41	0.10	0.28	1.14	1.22
	Interstitial B (Figure S33b)	-0.43	3.01	2.35	2.39	0.38	0.22	1.13	1.24
	Interstitial C (Figure S33d)	-0.34	3.04	2.39	2.38	-0.28	0.27	1.14	1.17
	Interstitial D (Figure S33c)	-0.30	3.11	2.43	2.37	0.07	0.05	1.11	1.38
	Substitutional (Figure S31b)	1.10	1.16	2.48	2.40	0.50	0.50	2.50	2.44
Mn-doped ZnSe	Interstitial A (Figure S32a)	-0.92	2.36	2.43	2.54	-0.71	-0.88	3.36	3.23
	Interstitial B (Figure S32b)	-0.82	2.38	2.50	2.54	-0.36	-0.48	4.18	3.50
	Interstitial C (Figure S32c)	-0.70	2.36	2.42	2.43	-0.54	-0.71	3.31	4.25
	Interstitial D (Figure S32d)	-0.26	2.38	2.47	2.54	-0.61	-0.51	3.55	3.50
	Substitutional (Figure S31c)	-1.08	-0.53	2.59	2.33	-0.48	-0.31	4.43	4.36

[a]The formation energy of the dopants (M = Co or Mn) in ZnSe quantum nanoribbons, calculated within the level of SCAN functional. The formation energy is defined as follows:

$$\Delta E_f = [\ E_{tot}(M@ZnSe–NH_3) - E_{tot}(ZnSe–NH_3) - \textstyle\sum_{i,m}(n_m\,\mu_m - n_i\mu_i)\ ] \qquad (S33)$$

where E_{tot} (M@ZnSe–NH_3), E_{tot} (ZnSe–NH_3), and μ_m (Co and Mn), and μ_i (Zn) are represents the total ground state energy of the doped structure, undoped structure, and chemical potential of metals (Co, Mn, and Zn), respectively.

[b]The formation energy of the dopants (M = Co or Mn) in the bulk ZnSe structure, calculated within the level of SCAN functional. The formation energy is defined as follows:

$$\Delta E_{\mathrm{f}} = [\, E_{\mathrm{tot}}(\mathrm{M@ZnSe}) - E_{\mathrm{tot}}(\mathrm{ZnSe}) - \textstyle\sum_{\mathrm{i,m}}(n_{\mathrm{m}}\,\mu_m - n_{\mathrm{i}}\mu_i)\,] \qquad \text{(S34)}$$

where E_{tot}(M@ZnSe), E_{tot}(ZnSe), and μ_m(Co and Mn), and μ_i(Zn) are the total ground state energy of the doped structure, undoped structure, and chemical potential of metals (Co, Mn, and Zn), respectively.

[c]The average distance between the dopants (M = Co or Mn) and the neighboring Se atoms in the ZnSe quantum nanoribbons.

[d]The average distance between the dopants (M = Co or Mn) and the neighboring Se atoms in the bulk ZnSe structure.

[e]The total charge transfer from the dopants (M = Co or Mn) in ZnSe quantum nanoribbons and their corresponding bulk configurations, determined through Bader charge population analysis. The effective charge was calculated using as follows:

$$Q_M = Z_M - q_{Bader} \qquad \text{(S35)}$$

where Z_M represents the number of valence electrons of the dopants (M = Co or Mn), and q_{Bader} represents the calculated Bader charge. The positive and negative signs indicate the direction of charge transfer (loss or acceptance) from or towards the dopants.

Table S4. The summary of calculation results for doped ZnSe nanoclusters.

Samples	Configuration	$\Delta E^{\mathrm{i}}_{\mathrm{f,cluster}}$ [a] (eV)	$\Delta l^{\mathrm{cluster}}_{\mathrm{M-Se}}$ [b] (Å)	$q^{\mathrm{cluster}}_{\mathrm{Bader}}$ [c] (e)	$\mu^{\mathrm{cluster}}_{\mathrm{B}}$
Co-doped ZnSe	Substitutional (Figure S34b)	1.74 (0.64)	2.41	0.13	2.24
Mn-doped ZnSe	Substitutional (Figure S34c)	0.63 (-2.01)	2.49	0.27	4.25

[a]The formation energy of the dopants (M = Co or Mn) on substitutional sites of ZnSe nanoclusters, computed within the level of PBE functional. The formation energy is defined as follows:

$$\Delta E_{\mathrm{f}} = [\, E_{\mathrm{tot}}(\mathrm{M@ZnSe{-}NH_3}) - E_{\mathrm{tot}}(\mathrm{ZnSe{-}NH_3}) - \sum_{\mathrm{i,m}}(n_{\mathrm{m}}\,\mu_m - n_{\mathrm{i}}\mu_i)\,] \qquad \text{(S33)}$$

where $E_{\mathrm{tot}}(\mathrm{M@ZnSe{-}NH_3})$, $E_{\mathrm{tot}}(\mathrm{ZnSe{-}NH_3})$, and μ_m(Co and Mn), and μ_i(Zn) are the total ground state energy of the doped structure, undoped structure, and chemical potential of metals (Co, Mn, and Zn), respectively. The values inside parenthesis obtained at poor limit condition of source chemical potentials of metals.

[b]The average distance between the dopants (M = Co or Mn) and the neighboring Se atoms in the ZnSe nanoclusters.

[c]The total charge transfer from the dopants (M = Co or Mn) sites in ZnSe clusters, determined through Bader charge population analysis. The effective charge was calculated using as follows:

$$Q_{\mathrm{M}} = Z_{\mathrm{M}} - q_{\mathrm{Bader}} \qquad \text{(S35)}$$

where Z_{M} represents the number of valence electrons of the dopants (M = Co or Mn), and q_{Bader} represents the calculated Bader charge. The positive and negative signs indicate the direction of charge transfer (loss or acceptance) from or towards the dopants.

References for the Supporting Information

[S1] J. Zeng, F. Bian, J. Wang, X. Li, Y. Wang, F. Tian, P. Zhou, *J. Synchrotron Radiat.* **2017**, *24*, 509.

[S2] G. Kresse, J. Hafner, *Phys. Rev. B* **1993**, *47*, 558.

[S3] G. Kresse, J. Furthmüller, *Phys. Rev. B* **1996**, *54*, 11169.

[S4] J. P. Perdew, K. Burke, M. Ernzerhof, *Phys. Rev. Lett.* **1996**, *77*, 3865.

[S5] S. Grimme, S. Ehrlich, L. Goerigk, *J. Comput. Chem.* **2011**, *32*, 1456.

[S6] S. Grimme, J. Antony, S. Ehrlich, H. Krieg, *J. Chem. Phys.* **2010**, *132*, 154104.

[S7] J. Paier, M. Marsman, K. Hummer, G. Kresse, I. C. Gerber, J. G. Ángyán, *J. Chem. Phys.* **2006**, *124*, 154709.

[S8] W. Tang, E. Sanville, G. Henkelman, *J. Phys.* **2009**, *21*, 084204.

[S9] M. Yu, D. R. Trinkle, *J. Chem. Phys.* **2011**, *134*, 064111.

[S10] C. Freysoldt, J. Neugebauer, *Phys. Rev. B.* **2018**, *97*, 205425.

[S11] C. Freysoldt, J. Neugebauer, A. M. Z. Tan, R. G. Hennig, *Phys. Rev. B.* **2022**, *105*, 014103.

[S12] M. Gajdoš, K. Hummer, G. Kresse, J. Furthmüller, F. Bechstedt, *Phys. Rev. B* **2006**, *73*, 045112.

[S13] A. Piróth, J. Sólyom **(2008)**. *Fundamentals of the Physics of Solids: Volume II: Electronic Properties*. Berlin Heidelberg: Springer.

[S14] P. Cudazzo, I. V. Tokatly, A. Rubio, *Phys. Rev. B* **2011**, *84*, 085406.

[S15] F. Hüser, T. Olsen, K. S. Thygesen, *Phys. Rev. B* **2013**, *88*, 245309.

[S16] L. Matthes, O. Pulci, F. Bechstedt, *New J. Phys.* **2014**, *16*, 105007.

[S17] L. Matthes, O. Pulci, F. Bechstedt, *Phys. Rev. B.* **2016**, *94*, 205408.

[S18] V. Wang, N. Xu, J.-C. Liu, G. Tang, W.-T. Geng, *Comput. Phys. Commun.* **2021**, *267*, 108033.

[S19] Y. Kumagai, F. Oba, *Phys. Rev. B* **2014**, *89*, 195205.

[S20] C. Freysoldt, J. Neugebauer, C. G. Van de Walle, *Phys. Rev. Lett.* **2009**, *102*, 016402.

[S21] C. Freysoldt, B. Grabowski, T. Hickel, J. Neugebauer, G. Kresse, A. Janotti, C. G. Van de Walle, *Rev. Mod. Phys.* **2014**, *86*, 253.

[S22] F. Oba, Y. Kumagai, *Appl. Phys. Express* **2018**, *11*, 060101.

[S23] D. Broberg, K. Bystrom, S. Srivastava, D. Dahliah, B. A. D. Williamson, L. Weston, D. O. Scanlon, G.-M. Rignanese, S. Dwaraknath, J. Varley, K. A. Persson, M. Asta, G. Hautier, *Npj Comput. Mater.* **2023**, *9*, 72.

[S24] M. Arrigoni, Spinney https://gitlab.com/Marrigoni/spinney (accessed Sep 23, 2023).

[S25] C.-W. Lee, N. U. Din, K. Yazawa, W. Nemeth, R. W. Smaha, N. M. Haegel, P. Gorai, *J. Appl. Phys.* **2024**, *135*, 155101.

[S26] J. Buckeridge, *Comput. Phys. Commun.* **2019**, *244*, 329.
